# HD2A and HD2C co-regulate drought stress response by modulating stomatal closure and root growth in Arabidopsis

**DOI:** 10.3389/fpls.2022.1062722

**Published:** 2022-11-23

**Authors:** Muhammad Sufyan Tahir, Jim Karagiannis, Lining Tian

**Affiliations:** ^1^ Department of Biology, University of Western Ontario, London, ON, Canada; ^2^ London Research and Development Centre, Agriculture and Agri-Food Canada, London, ON, Canada

**Keywords:** histone deacetylase, HD2 family, HD2A, HD2C, drought stress, root growth, GA2ox2

## Abstract

Histone deacetylase 2 (HD2) is a unique family of histone deacetylases (HDACs) in plants. Despite evidence that certain HD2 family HDACs play an important role in plant growth and stress response, the coordination of HD2s in these processes remains largely unknown. We found that HD2-type, HD2A and HD2C coordinate to play a role in drought stress response in Arabidopsis. We showed that the *hd2a*.*hd2c* double mutant (Mac16) exhibit decreased drought survival and increased water loss as compared to the single mutants, *hd2a* and *hd2c*. Gene expression analysis showed that the *ABI1* and *ABI2* genes were upregulated and *SLAC1* was downregulated which led to the modified stomatal functioning in the Mac16 as compared to the single mutants. Overexpression of *HD2A* and *HD2C* showed enhanced drought survival and decreased water loss. We also showed that the *GA2ox1* and *GA2ox2* genes, which are involved in the catabolism of bioactive gibberellic acids, were upregulated in the Mac16 as compared to the single mutants, which led to a decreased root growth in the Mac16. Furthermore, we showed that HD2A and HD2C can physically interact and increased genome-wide H3K9 acetylation was observed in the Mac16, compared to the single mutants. Overall, our investigation revealed that HD2A and HD2C coordinate to play a cumulative role in drought stress response and root growth in Arabidopsis.

## Introduction

Plants, because of their sessile lifestyle, have developed complex and sophisticated mechanisms to adapt and respond to different environmental stresses (Cutler et al., 2010). Among the various stresses that may be encountered by plants throughout their life cycle, drought is one of the most damaging environmental factors. Water accessibility is fundamental to plant growth, survival, and productivity (Rosegrant and Cline, 2003; Lesk et al., 2016). Plants attempt to withstand drought stress and tolerate unfavourable conditions by modifying their behaviour and undergoing a series of morphological, physiological, and biochemical changes at different stages of their growth and development. Drought stress stimulates the activation of different stress-responsive genes that encode different functional and regulatory proteins that are associated with gene regulatory networks (Fujita et al., 2011; Kim et al., 2015; Sah et al., 2016).

Epigenetic regulation plays a key role in different biological processes ranging from developmental scheduling and maintaining genome stability, to the regulation of various environmental stress responses (Kapazoglou and Tsaftaris, 2011; Luo et al., 2012a; Kim et al., 2015). Epigenetic regulation of gene expression is governed by nucleosomal core histone modifications and DNA methylation (Shinozaki et al., 2003; Urano et al., 2010). The basic nucleosome is comprised of an octamer of core histone proteins with two molecules of each of H2A, H2B, H3, and H4. The N-terminal lysine residues of histone H3 and H4 allow for different post-translational histone modifications, inculding acetylation, phosphorylation, methylation, sumoylation and ubiquitination (Kapazoglou and Tsaftaris, 2011; Zhou et al., 2013). These modifications of lysine residues act as a switch to activate or repress the expression of associated genes, and therefore offer a flexible mode of gene expression regulation in developmental programming and abiotic stress response (Kurdistani et al., 2004; Kouzarides, 2007; Tahir and Tian, 2021). Histone acetylation and deacetylation are dynamic reversible processes and are catalysed by histone acetyltransferases (HATs) and histone deacetylases (HDACs), respectively. HATs transfer the acetyl group to core histone lysine residues resulting in transcriptionally active euchromatin, whereas HDACs remove the acetyl group from the core histone lysine residues leading to transcriptional repression of associated genes (Liu et al., 2014). Plant HDACs have been classified into three different families based on their sequence homology to yeasts: the reduced potassium efficiency 3 (RPD3) family, the silent induced regulator 2 (SIR2) family, and the histone deacetylase 2 (HD2) family (Pandey et al., 2002; Luo et al., 2012a; Kim et al., 2015). The RPD3 and SIR2 family HDACs are homologous to yeast HDACs and are found throughout eukaryotes. (Pandey et al., 2002). The HD2 family HDACs share no sequence homology to yeast HDACs and are found only in plants and green algae (Ma et al., 2013; Bourque et al., 2016). The HD2 family, being unique to plants, is emerging as an important regulator in different aspects of plant development and plant response to different biotic and abiotic stresses (Tahir and Tian, 2021).

Present in the Arabidopsis genome are HD2A (AT3G44750), HD2B (AT5G22650), HD2C (AT5G03740), and HD2D (AT2G27840) *(* Dangl et al., 2001; Bourque et al., 2016 *)*. Both HD2A and HD2B were shown to interact with ASYMMETRIC LEAVES 1/2 (AS1, AS2) to regulate the expression and distribution of miRNA165 and miRNA166, which are known to control leaf morphology in Arabidopsis (Kidner and Martienssen 2004; Ueno et al., 2007). Expression of all the HD2 genes was downregulated under abscisic acid (ABA) or salt stress and upregulated under cold stress in Arabidopsis (Kuang et al., 2012; Luo et al., 2012c, To et al., 2011). *HD2A* and *HD2C* were strongly upregulated upon heat stress (Buszewicz et al., 2016). A DDB1-CUL4 Associated Factor (DCAF) protein, called HOS15 was shown to interact with HD2C with the help of HOS15 binding protein, POWERDRESS (PWR) to carry out the HD2C degradation *via* ubiquitination to activate cold-responsive (COR) genes in Arabidopsis (Zhu et al., 2008; Park et al., 2018; Lim et al., 2020). Overexpression of *HD2C* and *HD2D* caused the upregulation of many stress-related genes and resulted in an increased tolerance to different abiotic stresses (Sridha and Wu, 2006; Buszewicz et al., 2016; Han et al., 2016; Farhi et al., 2017).

Plants have developed various strategies to survive under drought stress. Of these, stomatal closure is one of the first lines of defence with respect to maintaining water status (Bharath et al., 2021). Limited water supply causes the accumulation of ABA in leaves which initiates a signalling cascade to induce stomatal closure by upregulating *SLAC1 via* SnRK2’s phosphorylation activity (Hopkins and Hüner, 2008). ABA-INSENSITIVE 1 and 2 (ABI1 and ABI2) are two PP2C-type protein phosphatases and are considered negative regulators of ABA-mediated signaling and inhibit the phosphorylation activity of SnRK2, thus negatively regulate the stomatal closure in Arabidopsis (Schweighofer et al., 2004).

Gibberellic acids (GAs) are known to induce germination and play a role in the elongation of endodermal cells to promote root growth in Arabidopsis (Ubeda-Tomás et al., 2009). Maintaining a dynamic homeostasis of bioactive GAs for normal root growth is an important phenomenon which is carried out by GA2-oxidase (GA2ox) enzymes. The GA2ox family includes GA2ox1, GA2ox2, GA2ox3, GA2ox4, and GA2ox6 and play a significant role in limiting the levels of bioactive GAs GA1 and GA4. Transcriptional activation of GA2ox genes is associated with histone acetylation status mediated by ABA in response to developmental signals and abiotic stresses including salt, drought, and cold (Rieu et al., 2008; Colebrook et al., 2014; Li et al., 2017; Chen et al., 2019).

HDACs generally do not function alone and are considered to play a role as a component of multiprotein complexes in a coordinated fashion. These complexes may include multiple HDACs, either belonging to the same family or different families (Chen and Wu, 2010; Luo et al., 2012c; Buszewicz et al., 2016; Li et al., 2017, Guo et al., 2020). HD2A, HD2C, and HD2D were shown to interact with HDA6 and HDA19, which are involved in the abiotic stress response (Luo et al., 2012c). The same HD2s were also shown to interact with DNMT2, a methyl transferase responsible for the methylation of DNA to mediate gene repression in Arabidopsis (Song et al., 2010). The association of different HD2s with each other during interaction with common interacting partners in response to different internal developmental and external environmental signals cannot be ruled out. Here, we show that HD2A and HD2C interacts to play a role in drought stress tolerance by regulating the expression of ABA-mediated stomatal closure-related genes. Both HD2s also play a cumulative role in regulating root growth by downregulating the expression of the GA2ox genes, which are thought to be involved in GA catabolism and degradation during osmotic stress in response to ABA-mediated stress signalling.

## Material and methods

### Phylogenetic analysis

Peptide sequences of all Arabidopsis HD2 genes were obtained from the Phytozome (https://phytozome-next.jgi.doe.gov/) and a phylogenetic tree was generated using MEGA X (Kumar et al., 2018). Amino acid sequence identities and divergences were determined using the National Center for Biotechnology Information (NCBI) tool BLAST for nucleotide sequences (https://blast.ncbi.nlm.nih.gov/Blast.cgi).

### Plant materials and growth conditions

*Arabidopsis thaliana* wild-type (WT) and T-DNA insertional mutants GK355-H03 (*hd2a*: At3g44750), SAIL1247-A02 (*hd2b*: At5g22650), SALK129799 (*hd2c*: At5g03740) and GK279-D04 (*hd2d:* At2g27840) were used in this study. All these genotypes were in accession Columbia (Col-0) background and have been used previously (Song et al., 2010; Buszewicz et al., 2016; Li et al., 2017). The T-DNA mutant lines were obtained from the Arabidopsis Biological Resource Center (ABRC). The double mutant lines used in this study were generated by crossing *hd2* single mutants with each other in different combinations. All the T-DNA insertion mutant lines were verified by genotyping by PCR using primers listed in **Supplementary Table 1**.

For plant growth experiments, all seeds were surface-sterilized in ethanol (70%) followed by bleach (25%) and stratified in the dark for 3 days at 4°C before sowing on ProMix-BX soil (Premier Horticulture, Québec) or on Murashige and Skoog (MS) growth medium (2.15 g/L) with or without additives such as antibiotics, plant growth regulators, or chemicals. Plants in the growth room were grown under constant 16 hours of daylight (long-day conditions) at 22°C with a relative humidity of 60%. Plants in the growth chambers were also grown under the same conditions, except dark cycles were at 18°C.

### Generation of plasmid constructs and transgenic plants

To generate HD2 overexpression (OE) vectors (CaMV35S:AtHD2), the full-length cDNA excluding the stop codon was amplified and cloned into the entry vector, pDONR221 (Invitrogen) using BP Clonase reaction mix (Thermo Fisher Scientific cat. 11789020) according to manufacturer’s protocol. The cloned vector was transferred into *Escherichia coli* strain DH5α. Subsequently, the cloned DNA was transferred from the entry vector to the destination vector, pEarleyGate101 (Hartley et al., 2000; Earley et al., 2006) using LR Clonase reaction mix (Thermo Fisher Scientific cat. 11791100) according to manufacturer’s protocol. The HD2-OE constructs were transferred to *Agrobacterium tumefaciens* strain GV3101 *via* electroporation (Wise et al., 2006). Primers used for gene cloning are listed in **Supplementary Table 2**. Plant transformation of WT Arabidopsis was carried out using the floral dip method (Zhang et al., 2006). The putative transgenic seeds were grown on selection medium (MS agar medium containing BASTA (glufosinate ammonium 25 mg/L) and two highly expressed transgenic lines for *HD2A* and *HD2C* were selected.

### Gene expression analysis

To study mRNA expression of different genes in *hd2* mutants and HD2 overexpression lines, quantitative real time PCR (RT-qPCR) was performed. RNA was isolated from leaves or seedlings using the Plant/Fungi Total RNA Purification Kit (Norgen) and cDNA was synthesized using iScript Reverse Transcription Supermix (Bio-Rad). The RT-qPCR was performed using a SsoFast EvaGreen Supermix kit (Bio-Rad) on CFX96 Real-time PCR detection system (Bio-Rad) following the manufacturer’s instructions. Data were analyzed using Bio-Rad CFX Manager 3.1 software. The expression levels were normalized to the *ACTIN2* gene. The ΔΔCT method was applied to calculate fold change in the expression level (Livak and Schmittgen, 2001). Three independent RT-qPCR analysis were performed with three technical replicates for each biological replicate. All primers used for RT-qPCR analysis are listed in **Supplementary Table 3**.

### Western blot analysis

Nuclei protein was extracted following the histone extraction protocol (abcam). Briefly, 10-day old seedlings were ground and fixed in Triton Extraction Buffer [TEB: PBS containing 0.5% Triton X-100, 2 mM phenylmethylsulfonyl fluoride (PMSF)]. After incubating the mixture on ice for 10 minutes, samples were centrifuged at 2000 rpm for 10 minutes at 4°C and the supernatant was discarded. TEB buffer (100 µl) was added to the samples and the centrifugation step was repeated. Cell pellets were resuspended in 0.4 µl of 0.2 N HCl and samples were kept at 4°C for 4 hours. Samples were then centrifuged at 2000 rpm for 10 minutes at 4°C. The supernatant was collected and the protein concentration was estimated using Bradford assay reagents (Thermo Fisher Scientific) following the standard protocol.

To separate proteins by SDS PAGE, samples were denatured by adding 20 mM dithiothreitol (DTT) and heating at 95°C for about 10 minutes. Samples were loaded onto SDS-PAGE gels (Bio Rad) to separate the proteins. Proteins from gels were transferred to PVDF membranes (Bio-Rad) using a Trans-Blot Semi-Dry Electrophoretic Transfer Cell (Bio-Rad). The PVDF membranes were then incubated overnight at 4°C in anti-H3K9ac or Anti-H3 antibody solution (Cell Signaling Technology and Millipore), followed by incubation in the secondary antibody (Milipore Sigma) solution for 1 hour. Proteins blots were detected using the EZ-ECL Chemiluminescence Detection Kit (Biological Industries) as per the manufacturer’s instructions with a MicroChemi imager (DNR Bio-Imaging Systems).

### Drought stress treatment

Soil drying or Polyethylene glycol (PEG-6000) methods were employed for plant drought treatment (Jiang et al., 1995; Landjeva et al., 2008). For the soil drought method, four-week old plants in the drought group were withheld from watering for 7 days to establish a drought stress. For PEG-6000, 10-day old seedlings were exposed to PEG (10%) in MS media for 24-72 hours before sample collection for gene expression analysis.

For the drought stress tolerance assay, two-week old *hd2* mutant or HD2-OE plants in drought-treated groups were withheld from watering for 11 and 13 days, respectively (Ge et al., 2018). After the drought stress period, plants were rewatered for two days to allow them to recover. Plants with at least two green turgid rosette leaves were considered to have survived. The survival rate for each genotype was determined based on the number of plants survived out of total number of plants grown.

### Leaf relative water content measurement

Leaf relative water content (RWC) was measured from plant leaves as previously described (Barrs and Weatherley, 1962). A total of three healthy rosette leaves were excised from the plants and fresh weight (FW) of all the collected leaves was determined immediately. All the leaves were rehydrated by floating them on water surface for 4 hours and leaf turgid weight (TW) was obtained. All the turgid leaves were oven-dried at 55°C for 24 hours. Oven-dried leaves in the tubes were weighed again to determine the leaf dry weight (DW). Leaf RWC (%) was determined as [(FW – DW)/(TW – FW)] x 100.

### Fresh leaf water loss measurement

To measure fresh leaf water loss in different genotypes, healthy leaves were detached from plants grown under control conditions and immediately weighed to measure their fresh weights. Leaves were weighed after every 30-minute interval for a total of 180 minutes. During each 30-minute interval, leaves were kept open in petri dishes at room temperature. Results were calculated as the percentage water loss compared to the leaf fresh weights.

### Stomatal aperture measurement

To examine stomatal opening and closing in *hd2* mutants and HD2-OE lines, stomatal aperture measurement assays were performed following the established protocol (Eisele et al., 2016; Scarpeci et al., 2017) with minor modifications. Briefly, healthy leaves were excised from plants and floated (with abaxial side down) in petri dishes containing MES/KOH stomatal opening buffer (10 mM MES, 5 mM KCl, 50 μM CaCl2, pH 6.15) for two hours and was used as control. For ABA treatment, the same steps were followed, except that, after two hours, 10 μM of ABA was added to the petri dishes containing MES/KOH buffer. After two hours of ABA treatment, leaves were taken out from the solution and quickly pat dried with napkins and prepared for microscopy. Preparation of leaf imprints to visualize stomata using the microscopy was performed by following the protocol described by Scarpeci et al. (2017) with minor modifications. Briefly, leaf imprints were obtained by applying a thin layer of transparent nail varnish on the abaxial leaf surface. The applied nail varnish layer was dried for about 30 minutes. The thin layer of nail varnish was transferred to a glass slide by gently pressing the leaf onto the slide. Stomata imprints were observed under a 40x objective lens using the EVOS™ XL Core Imaging System (AMEX1000). Stomatal pore widths and lengths were measured using the image processing software ImageJ (http://rsb.info.nih.gov/ij/). Results for stomatal aperture were shown as the ratio of width to length.

### Root growth phenotype analysis

Primary root lengths and secondary root number were examined in different genotypes under control and different treatments. For osmotic stress in seedlings in MS media, PEG-6000 was used (Jiang et al., 1995; Landjeva et al., 2008). Seeds of *hd2* mutants and HD2-OE lines were grown on agar plates containing only MS media or MS media supplemented with PEG-6000 (10%). For gibberellic acid treatments, GA3 and GA4 were added to the MS media at 1 μM and 10 μM concentrations. All the plates were placed vertically in the growth chamber to allow longitudinal root growth. Primary root length and number of secondary roots were examined on day 10 of germination for all the experiments.

### Yeast-two hybrid assay

To study the protein-protein interactions *in vivo*, yeast two-hybrid (Y2H) assays were performed following the instructions provided in the Matchmaker Gold Yeast Two-Hybrid System manual (Clontech) (Brückner et al., 2009). Using LR Clonase reaction mix, the cloned DNA from pDONR221 was transferred to destination vectors, pGBKT7 (bait vector), and the pGADT7 (prey vector). The Y2HGold strain was co-transformed with 5 µg of each of bait and prey vectors containing HD2 genes. For negative controls, the yeast strain was transformed with bait vector and empty prey vector (containing no gene of interest). The pGBKT7-53 and pGADT7-T plasmids provided with the kit (encode murine p53 and SV40 large T-antigen) were used as positive controls. The transformed Y2HGold cells were plated on minimal selective medium SD-Leu/Trp double dropout (DDO) and sub-cultured to selective medium SD-Leu/Trp/Ade/His quadruple dropout (QDO) and QDO supplemented with 40 mg/L X-Alpha Gal (5-Bromo-4-Chloro-3-indolyl a-D-galactopyranoside) and 200 µg/L Aureobasidin-A (QDO/X/A).

### Bimolecular fluorescence complementation assay

Bimolecular fluorescence complementation (BiFC) assays were performed to confirm the protein-protein interactions in a plant host (Tian et al., 2011). Using LR Clonase reaction mix, the cloned DNA from pDONR221 was transferred to destination vectors pEarleyGate201-YN and pEarleyGate201-YC to generate plasmid constructs for the BiFC assay. Both vectors were transferred to *Agrobacterium tumefaciens* strain GV3101 *via* electroporation. Agrobacterium cultures containing pEarleyGate201-HD2-YN and pEarleyGate201-HD2-YC constructs were co-infiltrated into *Nicotiana benthamiana* leaf epidermis. (Sparkes et al., 2006; Tian et al., 2011). For negative controls, pEarleyGate201-HD2-YN construct was co-infiltrated with an empty pEarleyGate202-YC vector. The YFP signal was observed after 48-72 hours using the Olympus Confocal Laser Scanning Microscope FV3000. The argon excitation laser wavelength was set at 514 nm to visualize the YFP signal.

### Statistical analysis

For statistical analysis, Microsoft Excel 2016 (Microsoft Corp., Washington, USA) was used to calculate simple univariate statistics, such as means, standard deviations, and standard errors. The statistical analyses were performed using IBM SPSS Statistics version 25.0 (IBM Corp. Armonk, New York). A p-value < 0.05 (*) or 0.01 (**) was used to show a statistically significant difference.

## Results

### HD2 family HDACs respond to drought stress in Arabidopsis

First, we performed phylogenetic and sequence alignment analysis of Arabidopsis HD2 proteins. Analysis showed that HD2A and HD2B are the closest relatives and share the highest sequence similarity (50.5%) and the least divergence (0.493) with each other at the protein level (**Supplementary Figure S1**). HD2A and HD2C shares the second highest similarity (40.5%) and the second least divergence (0.802), whereas HD2D is a distantly related member of the HD2 family and shares the least similarity and the maximum divergence with HD2A, HD2B and HD2C.

**Figure 1:**
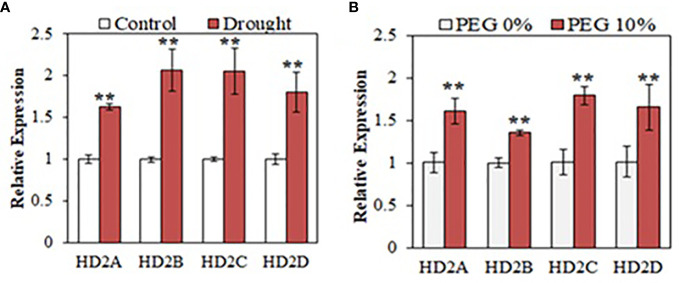
HD2 family HDACs respond to drought stress in Arabidopsis. Relative expression of HD2 family genes, *HD2A*, *HD2B*, *HD2C*, and *HD2D* under drought conditions **(A)** and PEG-6000-induced osmotic stress **(B)** in WT Arabidopsis. Data are shown as the expression levels relative to the control condition. Data shown are means ± standard errors (n = 3). The significance of the differences was determined by Student’s t test (**p < 0.01).

To examine how HD2 family genes respond to drought stress, we measured the expression levels of the HD2 family genes, *HD2A*, *HD2B*, *HD2C*, and *HD2D* under drought conditions. For soil drought, four-week old Arabidopsis wild-type (WT) plants were withheld from watering and leaf relative water content was measured to determine the water status of the plants (**Supplementary Figure S2A, B**). Leaf samples were collected daily from both the control group and the drought-treated group to examine gene expression by quantitative PCR (qPCR). The *Responsive to desiccation 29A* (*RD29A*) gene, which is induced under different abiotic stresses and frequently used as a drought responsive marker (Msanne et al., 2011; Bihmidine et al., 2013), was used to affirm the effectiveness of the drought treatment at the molecular level (**Supplementary Figure S2C**). *RD29A* showed significant upregulation starting on day 3 of drought treatment. When an effective drought stress initiating from day 3 was confirmed, we then examined the expression of *HD2A*, *HD2B*, *HD2C* and *HD2D* using samples from day 3 of the drought treatment. Analysis showed that all four HD2 genes were upregulated in response to drought (**Figure 1A**). Polyethylene glycol (PEG) is frequently used to mimic drought stress in plants and to study related responses (Jiang et al., 1995; Landjeva et al., 2008). To establish osmotic stress in MS media, seedlings were treated with PEG-6000 (10%) for 24 hours, and expression of HD2s was measured. Analysis showed that all HD2 genes were upregulated in response to PEG-induced osmotic stress (**Figure 1B**).

### Arabidopsis HD2A and HD2C positively regulate plant response to drought stress

To investigate the relationship of different HD2s in drought stress response, we used *hd2a*, *hd2b*, *hd2c*, and *hd2d* T-DNA insertional mutants in our study. Genotyping by PCR was done to identify homozygous mutant lines for each HD2 gene (**Supplementary Figure S3**). qPCR analysis was performed to confirm the knockout of HD2 gene expression at the mRNA level in *hd2* single mutants. Analysis showed that the expression of the *HD2A* and *HD2C* was completely knocked out in the *hd2a* and *hd2c* mutant lines, respectively, whereas *HD2B* and *HD2D* genes retained their expressions at 21% and 10% of the WT levels in the *hd2b* and *hd2d* mutant lines, respectively.

**Figure 2:**
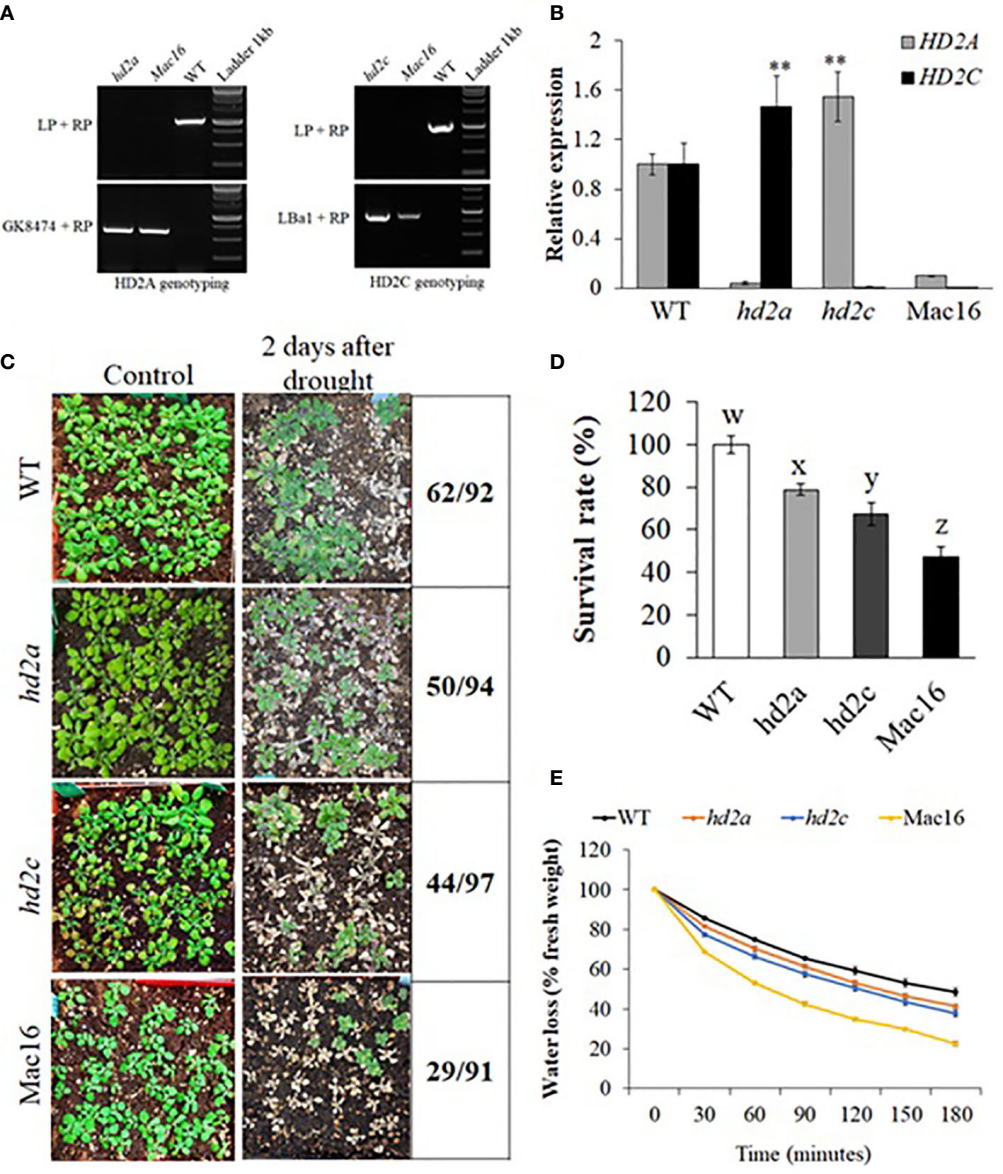
Confirmation of hd2 mutants and drought stress-induced changes in the hd2 mutant lines. **(A)** Genotyping of double mutant line Mac16 for *HD2A* and *HD2C* genes. Gene-specific left (LP) and right (RP) primers along with TDNA primers GK8474 and LBa1 were used in genotyping. **(B)** Relative expression of *HD2A* and *HD2C* in WT, the single mutants *hd2a* and *hd2c*, and the double mutant Mac16. Data shown are means ± standard errors (n = 3). (**p < 0.01). **(C)** Images of WT, *hd2a*, *hd2c*, and Mac16 plants under control and two days after rewatering, following 11 days of drought stress. **(D)** Survival rates of *hd2a*, *hd2c*, and Mac16 relative to WT plants after drought stress. Data shown are means ± standard errors (n = 3) from three independent experiments. In each experiment, at least 30 plants of each genotype were used to calculate the survival. The significance of the differences between different genotypes was determined by one-way ANOVA followed by post-hoc Tukey’s HSD tests. Lowercase letters indicate significant differences (p < 0.01). **(E)** Fresh leaf water loss measured in WT, *hd2a*, *hd2c*, and Mac16 at every 30-minute interval for the period of 180 minutes. Data shown are means ± standard errors (n = 3) from three independent experiments. In each experiment, measurements were taken from three plants (three leaves per plant).

HD2C has been shown to be involved in ABA and abiotic stress response and can mediate root growth under stress (Sridha and Wu, 2006; Luo et al., 2012c; Buszewicz et al., 2016; Chen et al., 2018). To study the relationship of HD2C with other HD2s, we crossed the *hd2c* mutant line with other *hd2* single mutants and successfully generated double mutant lines *hd2a.hd2c* (Mac16) and *hd2b*.*hd2c* (Mbc68). We could not obtain seeds for *hd2c*.*hd2d* double mutant line in several attempts of crossing *hd2c* with *hd2d*. The Mbc68 double mutant line was morphologically similar to the single mutants *hd2b* and *hd2c* and did not show any significant change in primary root length as compared to the respective single mutants *hd2b* and *hd2c* (**Supplementary Figure S4**). The Mac16 line was morphologically distinct from the respective single mutants *hd2a* and *hd2c* (**Supplementary Figure S5**). Due to the relative paucity of information on *hd2a*.*hd2c* double mutants, we focused our attention on the Mac16 (*hd2a*.*hd2c*) double mutant line along with *hd2a* and *hd2c* single mutants for further study. The homozygosity of T-DNA insertions in the double mutant line Mac16 was confirmed by PCR genotyping (**Figure 2A**). The qPCR analysis of *hd2* single and double mutants revealed that when the *HD2A* is knocked out, *HD2C* is upregulated in the *hd2a* mutant background. Similarly, when the *HD2C* is knocked out, *HD2A* is upregulated in the *hd2c* mutant line (**Figure 2B**). Next, we performed drought treatment assays to examine the survival of the single mutants *hd2a* and *hd2c* as well as the double mutant Mac16 under drought (**Figure 2C, D**). The single mutants *hd2a* and *hd2c* showed 79% and 67% survival, respectively, relative to WT. In contrast, the double mutant Mac16, showed only 47% survival relative to WT, which is also significantly lower than that observed in the single mutants. We also measured the fresh leaf water loss in these genotypes. The *hd2a* did not show significant change in the water loss, whereas the *hd2c* had increased water loss. The knockout of both *HD2A* and *HD2C* resulted in a significantly increased water loss in the Mac16 as compared to both single mutants and WT (**Figure 2E**, **Supplementary Figure S7**).

**Figure 3:**
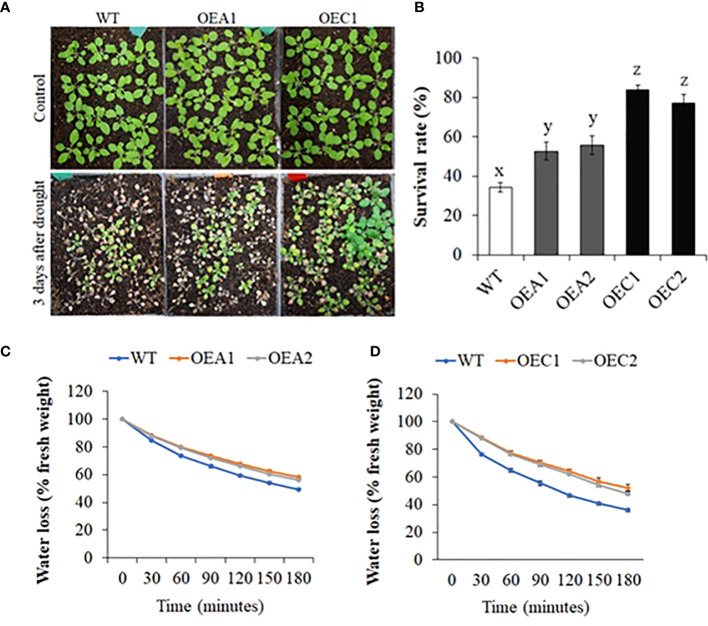
Drought survival and leaf water loss analysis in HD2 overexpression lines. **(A)** Images of WT and HD2-OE plants under control and two days after rewatering, following 13 days of drought stress. **(B)** Survival rates of WT, *HD2A*, and *HD2C* overexpression lines after drought stress. Data shown are means ± standard errors (n = 3) from three independent experiments. In each experiment, at least 30 plants of each genotype were used to calculate the survival. The significance of the differences between different genotypes was determined by one-way ANOVA followed by post-hoc Tukey’s HSD tests. Lowercase letters indicate significant differences (p < 0.01). **(C, D)** Fresh leaf water loss measured in HD2A-OE **(C)** and HD2C-OE **(D)** plants at every 30-minute interval for the period of 180 minutes. Data shown are means ± standard errors (n = 3) from three independent experiments. In each experiment, measurements were taken from three plants (three leaves per plant).

Along with the double mutant Mac16, we also generated *HD2A* (OEA1 and OEA2) and *HD2C* (OEC1 and OEC2) overexpression (OE) lines (**Supplementary Figure S6**) and studied whether *HD2A* or *HD2C* overexpression affects the survivability under drought stress (**Figures 3A, B**). While the WT plants showed 34% survival, the HD2A-OE lines, OEA1 and OEA2 showed increased survival of 52% and 56%, respectively, whereas HD2C-OE lines OEC1 and OEC2 showed 84% and 77% survival, respectively. Fresh leaf water loss analysis showed that both *HD2A* and *HD2C* overexpression lines had reduced water loss from the leaves as compared to WT (**Figures 3C, D**, **Supplementary Figure S7**).

### HD2A and HD2C co-regulate stomatal closure under stress

**Figure 4:**
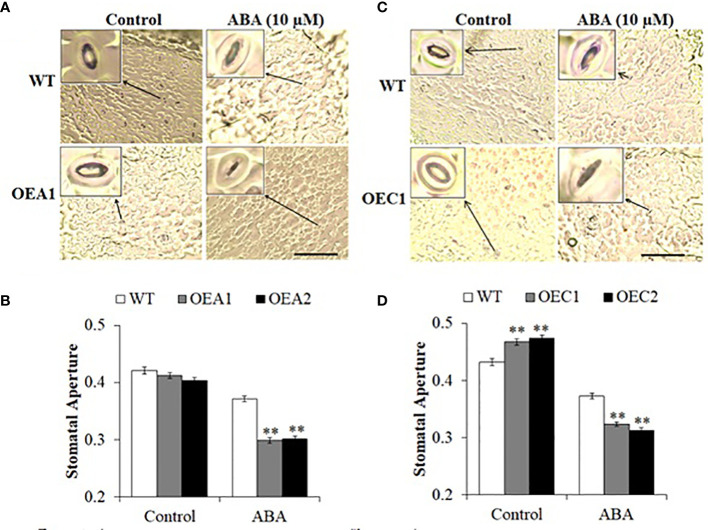
Stomatal closure under stress in HD2A and HD2C overexpression lines. Images of stomatal pore opening and closure observed in WT, HD2A-OE line **(A)** and HD2C-OE line **(C)** under control and ABA-induced stress (scale bar: 50 µm). **(B)** Stomatal aperture measured as width/length of stomatal pores in WT, HD2A-OE lines, OEA1 and OEA2 **(B)** and HD2C-OE lines, OEC1 and OEC2 **(D)** under control and ABA-induced stress. Data shown are means ± standard errors from at least 150 stomata. Three independent experiments were performed in this test. In each experiment, stomatal measurements were taken from three plants of each genotype (two leaves per plant). The significance of the differences between different genotypes was determined by Student’s t test (**p < 0.01).

**Figure 5:**
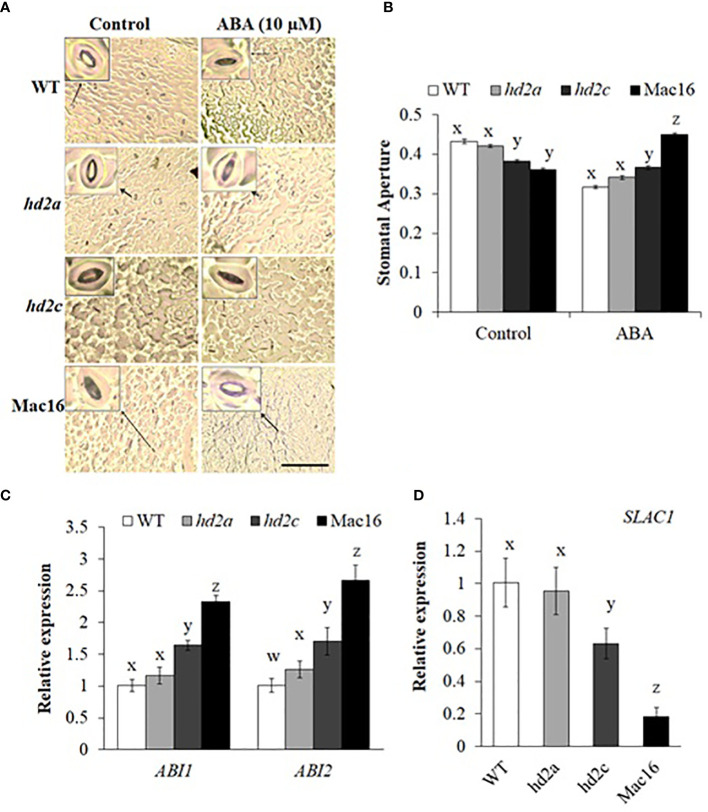
Stomatal closure and gene expression analysis under stress in hd2 mutant lines. **(A)** Images of stomatal pore opening and closure observed in WT, *hd2a*, *hd2c*, and Mac16 under control and ABA-induced stress (scale bar: 50 µm). **(B)** Stomatal aperture measured as width/length of stomatal pores in WT, *hd2a*, *hd2c*, and Mac16 under control and ABA-induced stress. Data shown are means ± standard errors from at least 150 stomata. Three independent experiments were performed in this test. In each experiment, stomatal measurements were taken from three plants of each genotype (two leaves per plant). **(C, D)** Relative expression of *ABI1* and *ABI2* **(C)** and *SLAC1* **(D)** in WT, *hd2a*, *hd2c*, and Mac16 under drought conditions. Data shown are means ± standard errors (n = 3). **(B-D)** The significance of the differences between different genotypes was determined by one-way ANOVA followed by post-hoc Tukey’s HSD tests. Lowercase letters indicate significant differences (p < 0.01).

Accumulation of ABA in leaves under drought stress initiates a signalling cascade to induce stomatal closure to minimize transpirational water loss (Hopkins and Hüner, 2008). Stomata are closed to limit water loss and establish an equilibrium between water absorbed by roots and water loss by transpiration (Wilkinson and Davies, 2002). We examined stomatal closure in HD2-OE and *hd2* mutant lines. The HD2A-OE lines did not show any differences in stomatal aperture under control conditions, whereas stomata closed completely under ABA-induced stress as compared to WT (**Figures 4A, B**). Stomata opened fully in HD2C-OE lines under control conditions and closed completely under ABA-induced stress as compared to WT (**Figures 4C, D**). Among the *hd2* mutants, *hd2a* did not show any change in stomatal aperture under control conditions, whereas the *hd2c* and Mac16 mutants showed reduced stomatal aperture with respect to WT (**Figures 5A, B**). Under ABA-induced stress, the *hd2c* mutant showed larger stomatal aperture as compared to the *hd2a* and WT. Knockout of both genes in the Mac16 resulted in a significantly larger stomatal aperture as compared to both single mutants and WT. This suggests that HD2A and HD2C corelate in regulating stomatal closure to play a role in the drought stress response.

Accumulation of ABA in leaves under drought stress stimulates a core signalling pathway to induce stomatal closure by upregulating the expression of *SLAC1* gene (Vahisalu et al., 2008; Munemasa et al., 2015; Zhang et al., 2016), whereas ABI1 and ABI2, the two protein phosphatases, are considered negative regulators of ABA-mediated stomatal closure. We examined the expressions of *ABI1*, *ABI2*, and *SLAC1* genes in *hd2* mutants under drought stress. Analysis of *ABI1* and *ABI2* gene expression (**Figure 5C**) showed that their expression was not changed in the *hd2a* mutant, while the *hd2c* mutant exhibited an upregulation of both genes. Knockout of both *HD2A* and *HD2C* in Mac16 resulted in a significant upregulation of *ABI1* and *ABI2* under drought stress as compared to the single mutants and WT. Gene expression analysis of *SLAC1* (**Figure 5D**) showed that its expression was not affected in *hd2a* but decreased in the *hd2c*. Knockout of both *HD2A* and *HD2C* in Mac16 resulted in a drastic decrease of *SLAC1* gene expression. Taken together, the HD2A and HD2C play a collective role in regulating stomatal opening and closure. Knockout of both genes led to a stronger phenotype in terms of drought tolerance and stomatal closure as compared to the single mutants, indicating an additive effect of HD2A and HD2C in drought stress response.

### HD2A and HD2C play a role in regulating root growth

**Figure 6:**
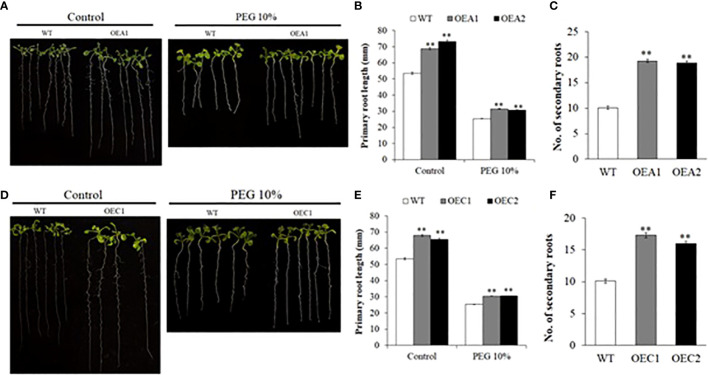
Root growth in HD2-OE lines under control and osmotic stress. Images of root growth in 10-day old WT, HD2A-OE **(A)** and HD2C-OE **(D)** seedlings under control and PEG-6000 (10%) conditions. **(B, E)** Primary root lengths measured in 10-day old WT, HD2A-OE **(B)** and HD2C-OE **(E)** seedlings under control and PEG (10%). **(C, F)** Secondary root number counted in 10- day old seedlings of WT, HD2A-OE **(C)** and HD2C-OE **(F)** seedlings under control conditions. Data shown are means ± standard errors (n = 60). Three independent experiments were performed in this test. In each experiment, 20 seedlings of each genotype were used to measure the primary root lengths. The significance of the differences between different genotypes was determined by Student’s t test (**p < 0.01).

**Figure 7:**
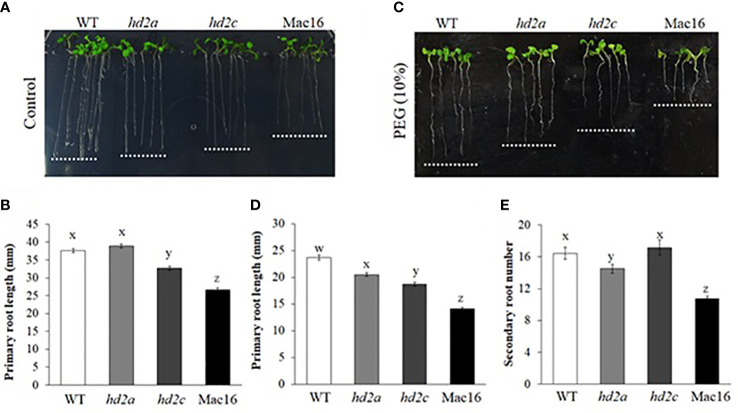
Root growth in hd2 mutants under control and osmotic stress. Images of root growth in 10-day old WT, *hd2a*, *hd2c*, and Mac16 seedlings under control **(A)** and PEG-6000 (10%) conditions **(C)**. **(B, D)** Primary root lengths measured in 10-day old WT, *hd2a*, *hd2c*, and Mac16 seedlings under control **(B)** and PEG-6000 **(D)** conditions. **(E)** Secondary root number counted in 10-day old WT, *hd2a*, *hd2c*, and Mac16 seedlings under control conditions. Data shown are means ± standard errors (n = 60). Three independent experiments were performed in this test. In each experiment, 20 seedlings of each genotype were used to measure the primary root lengths or secondary root numbers. The significance of the differences between different genotypes was determined by one-way ANOVA followed by post-hoc Tukey’s HSD tests. Lowercase letters indicate significant differences (p < 0.05).

Roots also play a vital role in the strategy of plant response to drought. Our next objective was to study if HD2A and HD2C play a role in regulating root growth under drought stress. First, we measured the primary and secondary root growth of HD2-OE and *hd2* mutant lines under control and drought stress conditions mimicked by PEG-6000. Overexpression of both *HD2A* and *HD2C* resulted in increased primary root length under control and osmotic stress conditions (**Figure 6**). These overexpression lines also showed a higher number of secondary roots under control conditions. Conversely, primary root length was significantly decreased in the Mac16 (71%) and *hd2c* (87%) mutants under control conditions as compared to WT (**Figures 7A, B**), whereas root length did not change in the *hd2a* mutant. Application of osmotic stress (PEG) caused a significant decrease in the primary root lengths of both *hd2a* (86%) and *hd2c* (79%) and a further decrease in the Mac16 (59%) as compared to WT (**Figures 7C, D**). Regarding the secondary root number in *hd2* mutants under control conditions, the *hd2c* did not show difference in the number of secondary roots, whereas the *hd2a* showed fewer secondary roots in comparison to WT. Knockout of both HD2A and HD2C resulted in a significant decrease in the number of secondary roots in the Mac16 line (**Figure 7E**).

### HD2A and HD2C decrease H3K9 acetylation and co-regulate GA2ox gene expression to maintain root growth

**Figure 8:**
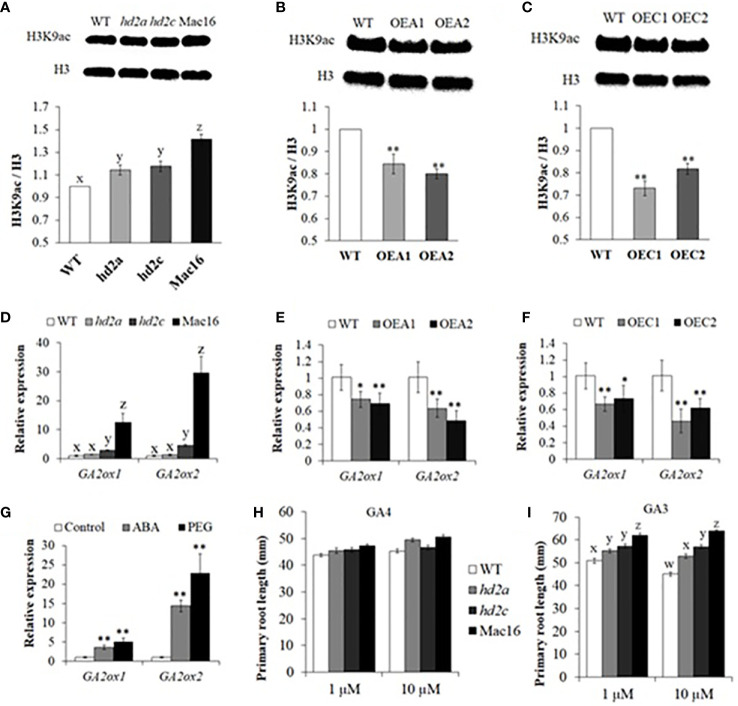
HD2A and HD2C decrease H3K9 acetylation level and coregulate root growth via GA2ox-mediated GA catabolism. **(A-C)** Western blots and relative intensities of H3K9ac/H3 in *hd2* mutants **(A)**, HD2A-OE **(B)**, and HD2C-OE **(C)** lines. The fold changes in the relative intensities of H3K9ac/H3 were normalized to WT. The intensities of blotting signals were quantified using Image J software. Data shown are means ± standard errors (n = 3) from three independent experiments. The significance of the differences between different genotypes was determined by one-way ANOVA followed by post-hoc Tukey’s HSD tests **(A)** and Student’s t test **(B-C)**. Lowercase letters or asterisks indicate significant differences at p < 0.01. **(D-G)** Relative expression of *GA2ox1* and *GA2ox2* genes in *hd2* mutants **(D)**, HD2A-OE **(E)**, and HD2C-OE **(F)** lines in 10-day old seedlings under control conditions. **(G)** Relative expression of *GA2ox1* and *GA2ox2* genes in WT under control, ABA (100 µM), and PEG-6000 conditions. Data shown are means ± standard errors (n = 3) from three independent experiments. The significance of the differences between different genotypes in **(D)** was determined by one-way ANOVA followed by post-hoc Tukey’s HSD tests. The significance of the differences between different genotypes in **(E-G)** was determined by Student’s t test. Lowercase letters indicate significant differences at p < 0.01, whereas asterisks indicate significant differences at *p < 0.05, **p < 0.01. **(H, I)** Primary root lengths measured in 10-day old WT, *hd2a*, *hd2c*, and Mac16 seedlings under GA4 (1 µM and 10 µM) **(H)** and GA3 (1 µM and 10 µM) **(I)** treatments. Data shown are means ± standard errors (n = 60). Three independent experiments were performed in this test. In each experiment, 20 seedlings of each genotype were used to measure the primary root lengths. The significance of the differences between different genotypes was determined by one-way ANOVA followed by post-hoc Tukey’s HSD tests. Lowercase letters indicate significant differences (p < 0.01).

The GA2ox enzymes play an important role in maintaining a dynamic homeostasis of bioactive GAs for normal plant root growth. We thus investigated whether the HD2A and HD2C can regulate the expression of the GA2ox genes. First, we performed western blot to examine global H3K9 acetylation levels in the *hd2* mutants and HD2-OE lines. The H3K9ac/H3 levels were found to be significantly higher in the Mac16 (1.41) as compared to the *hd2a* (1.14), *hd2c* (1.18), and WT (**Figure 8A**). Conversely, lower levels of H3K9ac/H3 were found in both *HD2A* and *HD2C* overexpression lines (**Figures 8B, C**). Next, we examined the expression of GA2ox family genes in *hd2* mutants and HD2-OE lines under control conditions. Expression of the *GA2ox1* and *GA2ox2* was upregulated many fold in the Mac16 as compared to the single mutants and WT (**Figure 8D**). The *hd2a* mutant did not show change in the expression of both genes. On the other hand, *HD2A* and *HD2C* overexpression caused a significant downregulation of both *GA2ox1* and *GA2ox2* (**Figures 8E, F**). Other GA2ox genes (*GA2ox3*, *GA2ox4*, *GA2ox6*) did not show significant changes in their expression levels. Previously, it has been shown that the transcriptional activation of GA2ox genes is associated with histone acetylation status triggered by ABA in response to internal developmental signals and abiotic stress conditions (Colebrook et al., 2014; Li et al., 2017; Chen et al., 2019). We analysed the expression of the *GA2ox1* and *GA2ox2* in WT under ABA and PEG treatments (**Figure 8G**). Analysis showed that the expression of both *GA2ox1* and *GA2ox2* was significantly upregulated in response to ABA and PEG. *GA2ox2* showed many fold higher expression as compared to the *GA2ox1*. These results indicate that *GA2ox1* and *GA2ox2* are upregulated in drought stress response *via* ABA pathway and their expression is co-regulated by HD2A and HD2C, possibly through histone acetylation modification in response to ABA and osmotic stress.

Bioactive gibberellic acid GA4 serves as a substrate for GA2ox-mediated GA catabolism and GA3 is not considered its substrate. Upregulation of GA2ox genes leads to the lower levels of GA4 and GA1 in Arabidopsis (Yamauchi et al., 2007; Rieu et al., 2008; Li et al., 2017). We thus examined the primary root lengths in *hd2* mutants under GA4 and GA3 treatments. The *hd2* mutant lines did not show any difference in root phenotype under 1 μM of GA4 treatment, with a slight increase in root lengths under 10 μM of GA4 (**Figure 8H**). However, GA3 treatments (1 μM and 10 μM) in *hd2* mutants led to a significant increase in primary root lengths of Mac16 as compared to the *hd2a*, *hd2c*, and WT (**Figure 8I**).

### HD2A and HD2C physically interact with each other

**Figure 9:**
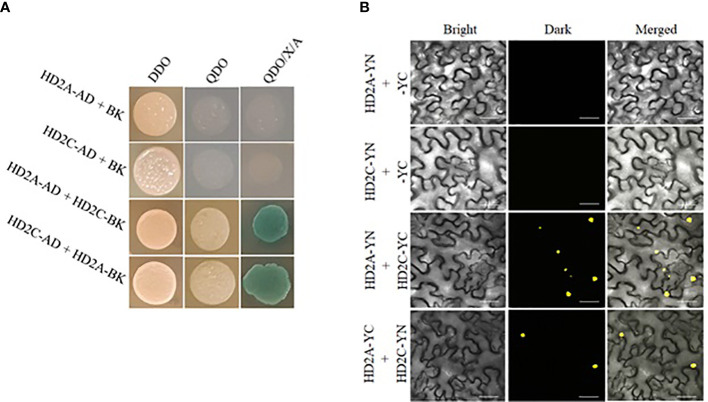
The HD2A and HD2C physically interact with each other. **(A)** Y2H assays showing interactions of HD2A and HD2C at the protein level. Transformed yeast cells were plated on minimal selective medium DDO (double dropout) and sub-cultured to QDO (quadruple dropout) and QDO/X/A (QDO supplemented with X-alpha galactosidase and Aureobasidin-A) medium to determine their ability to grow to identify protein interactions. Empty vector pGBKT7 was co-transformed with either HD2A-AD or HD2C-AD as a negative control. Blue colonies indicate protein-protein interaction. Three independent experiments were performed in this test. **(B)** BiFC (Bimolecular fluorescence complementation) assays showing interactions of HD2A and HD2C in the plant host. The constructs HD2A-YN and HD2C-YC (or HD2A-YC and HD2C-YN) were co-infiltrated into leaf epidermal cells of *Nicotiana benthamiana* via for transient expression. YFP (Yellow Fluorescent Protein) signal was detected after 48-72 hours of infiltration. Empty vector pEarleyGate202-YC was co-transformed with either HD2A-YN or HD2C-YN as a negative control. Three independent experiments were performed in this test. (scale bar 50 µm).

The additive effect of HD2A and HD2C in drought stress response and root growth led us to investigate if HD2A and HD2C can physically interact with each other. To study this protein-protein interaction, we performed yeast two-hybrid (Y2H) and bimolecular fluorescent complementation (BiFC) assays. In Y2H assays, the yeast strains containing constructs HD2A*-*AD or HD2C-AD along with an empty vector (pGBKT7) did not grow on selective medium QDO and QDO/X/A. However, when co-transformed with constructs expressing HD2A*-*AD and HD2C*-*BK (or HD2A*-*BK and HD2C*-*AD), yeast cells developed colonies on QDO and QDO/X/A, indicating a physical interaction of the HD2A and HD2C (**Figure 9A**). To further confirm this protein interaction in the plant host, BiFC assays were performed. The HD2A-YN or HD2C-YN construct did not show any YFP signal (yellow fluorescence) when co-transformed with an empty pEarleyGate202-YC vector. YFP signal in leaves was observed when both HD2A-YN and HD2C-YC constructs (or HD2A-YC and HD2C-YN) were infiltrated together, again indicating a physical interaction of HD2A and HD2C (**Figure 9B**).

## Discussion

In this study, we demonstrated that HD2A and HD2C positively regulate the plant response to drought stress and coordinate to regulate the expression of genes involved in ABA-mediated stomatal closure and GA-mediated root growth under drought stress. First, we showed that all Arabidopsis HD2 genes were significantly upregulated in response to the initial stage of drought stress (**Figure 1**). The upregulation of the HD2 family genes indicate that all members of HD2 family may be involved in the drought stress response. Previously, Arabidopsis plants treated with cold temperature showed the upregulation of the *HD2A*, *HD2B* and *HD2C* (To et al., 2011). Similarly, *HD2A* and *HD2C* were also shown to be upregulated under heat stress (Buszewicz et al., 2016). However, salt and ABA treatments resulted in the downregulation of HD2 genes in Arabidopsis (Luo et al., 2012c). Additionally, we demonstrated that both HD2A and HD2C positively regulate the drought stress response. Overexpression of *HD2A* or *HD2C* resulted in enhanced plant survival under drought and showed decreased water loss (**Figure 3**). On the other hand, the single mutants *hd2a* and *hd2c* showed lower plant survival under drought stress and showed increased water loss. Knockout of both *HD2A* and *HD2C* resulted in a significantly lower survival and increased water loss in the Mac16 as compared to the *hd2a* and *hd2c* (**Figure 2**). Previously, HD2C has been shown to be involved in ABA and abiotic stress responses. Overexpression of *HD2C* showed increased tolerance to ABA and salt (Sridha and Wu, 2006). Luo et al. (2012c) reported that the double mutant line *hd2c*.*hda6* had decreased tolerance to ABA and salt stresses as compared to the single mutants *hd2c* and *hda6*.

Under restricted water supply, stomata are closed to minimise water loss by transpiration. We investigated if decreased survival and increased water loss in *hd2* mutants is related to the functioning of stomata. Overexpression of *HD2A* or *HD2C* caused stomata to close completely in response to ABA (**Figure 4**). Whereas the Mac16 showed significantly larger stomatal aperture (i.e., stomata did not close) under ABA as compared to both single mutants (**Figures 5A, B**), suggesting that the decreased survival of the Mac16 under drought stress is related to increased water loss from the leaves due to modified stomatal functioning. ABA-mediated signalling causes the upregulation of *SLAC1 via* SnRK2/OST1 phosphorylation to initiate stomatal closure under drought stress (Zhang et al., 2016; Bharath et al., 2021). ABI1 and ABI2 are considered negative regulators of ABA-mediated stomatal closure and inhibit the expression of the *SLAC1* by dephosphorylating and subsequently down-regulating SnRK2/OST1 (Vahisalu et al., 2008; Roelfsema et al., 2012; Munemasa et al., 2015; Zhang et al., 2016). We thus analysed the expression of *ABI1*, *ABI2*, and *SLAC1* in *hd2* mutants under drought stress. Although the *hd2a* mutant did not show any significant change in the expression of the *ABI1* and *ABI2*, both gene were significantly upregulated in the *hd2c* mutant. Knockout of both *HD2A* and *HD2C* caused the further elevation of both *ABI1* and *ABI2* genes in the double mutant Mac16 as compared to the single mutants (**Figure 5C**). Lower expression of the HD2A and HD2C genes corresponds to the higher expression of *ABI1* and *ABI2*. Furthermore, the expression of the *SLAC1* was not affected in the *hd2a* and decreased significantly in the *hd2c* with a further decrease observed in the Mac16 as compared to the *hd2c* (**Figure 5D**). The higher expression of *ABI1* and *ABI2* corresponds with the lower expression of the *SLAC1*, resulting in the modification of stomatal opening and closing pattern in the Mac16 under stress. Moreover, higher H3K9 acetylation levels were observed in the Mac16 as compared to the single mutants (**Figure 8A**). Regulation of *ABI1* and *ABI2* gene expression is associated with the status of histone acetylation (Luo et al., 2012c). Our findings indicate that HD2A and HD2C coordinate to regulate the drought stress by modulating the expression of the *ABI1*, *ABI2*, and *SLAC1*, thus leading to ABA-mediated stomatal closure under drought stress.

Plants deal with the drought stress mainly in two organs including roots (to absorb water) and leaves (to retain water). Stomatal closure is one of the initial responses of plants to drought stress to maintain water status (Bharath et al., 2021). Persistent drought conditions cause the plants to modify root growth system to enhance their capability of absorbing soil water (Rosales et al., 2019). Water uptake by a plant is dependent on its root system architecture, which is determined by primary root growth and branching (Brunner et al., 2015; Rosales et al., 2019). We investigated whether the HD2A and HD2C play a role in regulating root growth under drought stress. Increased primary root length was observed in both *HD2A* and *HD2C* overexpression lines under control and osmotic stress conditions. These overexpression lines also displayed more secondary roots (**Figure 6**). Conversely, primary root length was significantly decreased in the Mac16 as compared to the single mutants under control conditions (**Figures 7A, B**). Application of osmotic stress (PEG) caused a significant decrease in primary root lengths of both *hd2a* and *hd2c*, and it was further decreased to a larger extent in the Mac16 under PEG stress (**Figures 7C, D**). For secondary roots, no difference was observed in the *hd2c*, whereas the *hd2a* showed lower number of secondary roots, compared to WT. However, knockout of both *HD2A* and *HD2C* resulted in a significant decrease in the number of secondary roots in Mac16 (**Figure 7E**). Li et al. (2017) reported that the double mutant *hd2a*.*hd2b* showed shorter roots as compared to the *hd2a* and *hd2b*. Overexpression of the *HD2C* showed increased root lengths under ABA and salt treatments, whereas the *hd2c* mutant line displayed shorter root lengths (Sridha and Wu, 2006).

The GA2ox enzymes play a significant role in maintaining a dynamic homeostasis of bioactive GAs for normal plant root growth. Histone acetylation modifications are well correlated with gene transcriptional regulation in plant abiotic stress response (Yuan et al., 2013; Chen et al., 2020). Transcriptional activation of GA2ox genes is associated with the modification of histone acetylation status triggered by different abiotic stresses (Rieu et al., 2008; Liu et al., 2010; Colebrook et al., 2014; Lee et al., 2016; Ravindran et al., 2017; Chen et al., 2019). Previously, knockout of both *HD2A* and *HD2B* were shown to cause the hyperacetylation of histone H3 at *GA2ox2* gene locus, resulting in the upregulation of the *GA2ox2*, which lead to shorter root lengths in the double mutant *hd2a*.*hd2b* (Li et al., 2017). In this study, we found higher H3K9 acetylation levels in the double mutant Mac16 as compared to the *hd2a* and *hd2c*, whereas decreased H3K9 acetylation levels were detected in the *HD2A* and *HD2C* overexpression lines (**Figures 8A, C**). Furthermore, expression of *GA2ox1* and *GA2ox2* was significantly upregulated in the Mac16 as compared to the single mutants (**Figure 8D**) and downregulated in both *HD2A* and *HD2C* overexpression lines (**Figures 8E, F**). We also showed that the expression of *GA2ox1* and *GA2ox2* was upregulated in response to ABA and PEG treatments in WT (**Figure 8G**). This indicates that the GA2ox genes are activated in response to stress signals to control root growth *via* GA catabolism. ABA accumulation under drought stress causes the upregulation of GA2ox genes *via* histone acetylation modification which leads to the lower levels of bioactive GAs (Footitt et al., 2011; Lee et al., 2016; Liu and Hou, 2018). Bioactive gibberellic acid GA4 serves as a substrate for GA2ox-mediated GA catabolism, whereas GA3 is not considered a substrate (Yamauchi et al., 2007; Rieu et al., 2008; Li et al., 2017). Yamauchi et al. (2007) reported that the *ga2ox2* mutant plants had higher levels of GA4. Moreover, GA4 has been shown to enhance root and shoot lengths (Cowling et al., 1998). We examined the primary root lengths in *hd2* mutants under GA4 and GA3 treatments. The *hd2* mutant lines did not show any differences in the primary root lengths under 1 μM of GA4. However, 1 μM of GA3 treatment led to a significant increase in the primary root lengths of Mac16 as compared to the *hd2a* and *hd2c* (**Figure 8I**). Taken together, higher levels of H3K9 acetylation correlates with the higher expression of the GA2ox genes in the *hd2* single and double mutants. Higher expression of the GA2ox genes might be associated with the lower levels of GA4, thus leading to decreased root growth in the *hd2* double mutant. GA3 is not considered as a substrate of GA2ox enzymes, thus it enhanced root growth in the *hd2* mutants.

The deacetylase activity of HDACs often depends on interaction and coordination with other HDACs and transcription factors (Yang and Seto, 2008). HD2A, HD2B and HD2C were shown to interact with DNMT2, a methyl transferase responsible for the methylation of DNA to mediate gene repression (Song et al., 2010). HD2A, HD2C, and HD2D were also shown to interact with HDA6 and HDA19, which are well known for their role in the abiotic stress response. Functional association within the HD2 family members as well as with the RPD3-type HDACs as part of repression complexes might be of vital importance for regulating gene expression through histone modifications (Tahir and Tian, 2021). Here, we investigated whether HD2A and HD2C can physically interact with each other. The Y2H assays demonstrated that both HD2s physically interact with each other. The BiFC assays in plant further confirmed the interaction of these two HD2s (**Figure 9**). Physical interaction between HD2s could be important to play a collective role in regulating target gene expression, thus mediating the drought stress response and root growth regulation in Arabidopsis.

**Figure 10:**
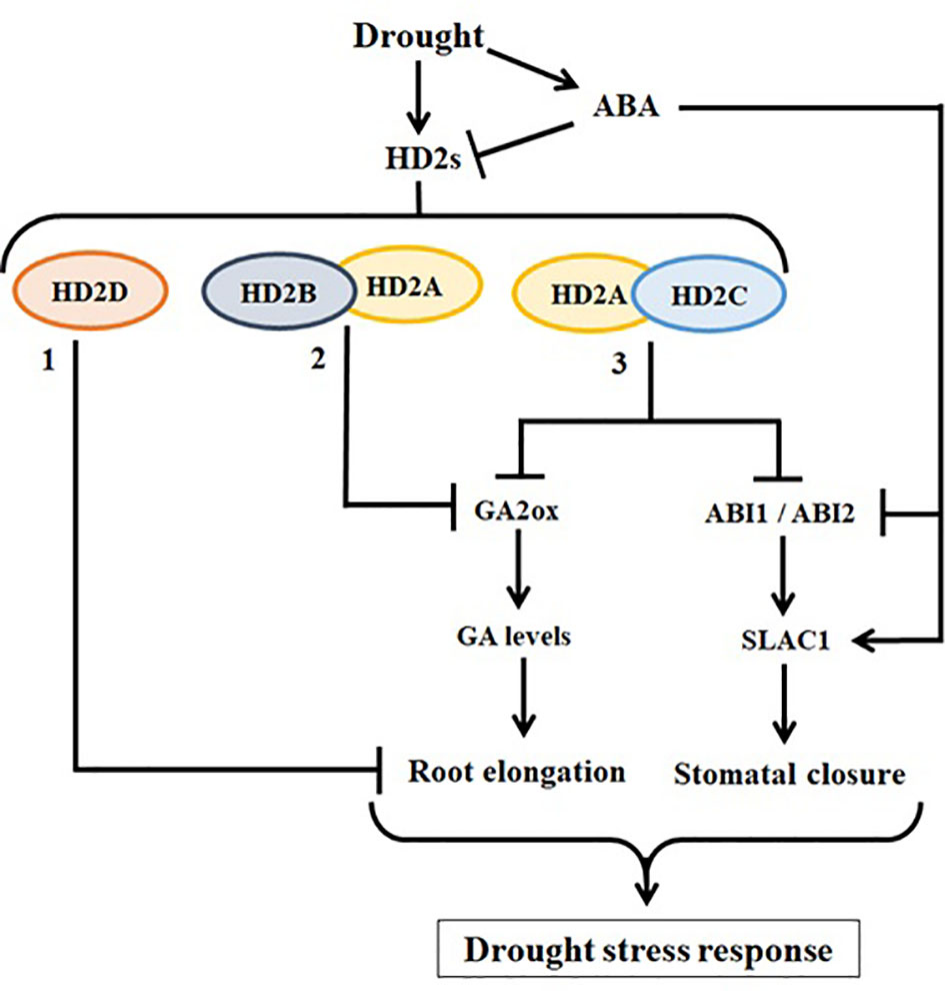
The working model of HD2 family HDACs involved in drought stress response in Arabidopsis. (1: Han et al., 2016) HD2D was reported previously as a negative regulator of primary root length. Overexpression of *HD2D* caused a decrease in primary root length and increase in the number of secondary roots, and HD2D-OE plants showed enhanced tolerance to drought stress. (2: Li et al., 2017) HD2A and HD2B were shown to negatively coregulate the expression of *GA2ox2* via histone acetylation modification, leading to decreased primary root growth in *hd2a*.*hd2b* double mutant. GA2ox genes are involved in the catabolism of bioactive gibberellins. (3) Our study showed that HD2A and HD2C coordinate to play role at both leaf and root levels by modulating the expression of *ABI1* and *ABI2*, involved in ABA-mediated stomatal closure, and *GA2ox* genes. The knockout of both HD2A and HD2C showed abnormal stomatal functioning and decreased root growth in *hd2a*.*hd2c* double mutant, leading to decreased drought stress response. All arrow heads represent positive regulation, while all stop lines represent negative regulation associated with related pathways.

It is evident that all HD2 genes are involved in abiotic stress response, indicating that HD2 proteins may not be redundant, but are functionally either independent or coordinative and/or complementary. These HD2s may follow different pathways to accomplish the same functions. The working model of HD2-type HDACs illustrated in **Figure 10** represents the involvement and coordination of HD2s in drought stress response. Li et al. (2017) reported that HD2A and HD2B co-regulate plant root growth by mediating the expression of GA2ox genes. Han et al. (2016) reported that the overexpression of the *HD2D* in Arabidopsis resulted in enhanced tolerance to drought stress. Currently, it is not known whether HD2D functions alone or coordinates with other HD2s in this regard. Our study showed that HD2A and HD2C coordinate to control water loss from leaves by controlling the stomatal closure *via* ABA signalling pathway of *SLAC1* gene regulation. Additionally, we showed that both HD2A and HD2C coordinate to downregulate the expression of the GA2ox genes to control root growth under drought. Thus, HD2A and HD2C play a role in the plant at both leaf and root levels to adopt a comprehensive strategy in responding to drought stress.

## Data availability statement

The original contributions presented in the study are included in the article/**Supplementary Material**. Further inquiries can be directed to the corresponding author.

## Author contributions

MT and LT conceived the research idea. MT designed the research, performed experiments, analyzed data, and wrote manuscript. JK and LT participated in designing the research. LT and JK organized and revised the manuscript. All authors contributed to the article and approved the submitted version.

## Acknowledgments

This research was supported by a Discovery Grant from the Natural Sciences and Engineering Research Council (NSERC).

## Conflict of interest

The authors declare that the research was conducted in the absence of any commercial or financial relationships that could be construed as a potential conflict of interest.

## Publisher’s note

All claims expressed in this article are solely those of the authors and do not necessarily represent those of their affiliated organizations, or those of the publisher, the editors and the reviewers. Any product that may be evaluated in this article, or claim that may be made by its manufacturer, is not guaranteed or endorsed by the publisher.
