# Supplementary material for: HD2A and HD2C co-regulate drought stress response by modulating stomatal closure and root growth in Arabidopsis
Source: Front Plant Sci. 2022 Nov 23;13:1062722. doi: 10.3389/fpls.2022.1062722 (PMC9727301; doi:10.3389/fpls.2022.1062722)
Supplement: Supplementary file 1 [file DataSheet_1.docx]

**Supplementary File 1 – Figures 1-7**


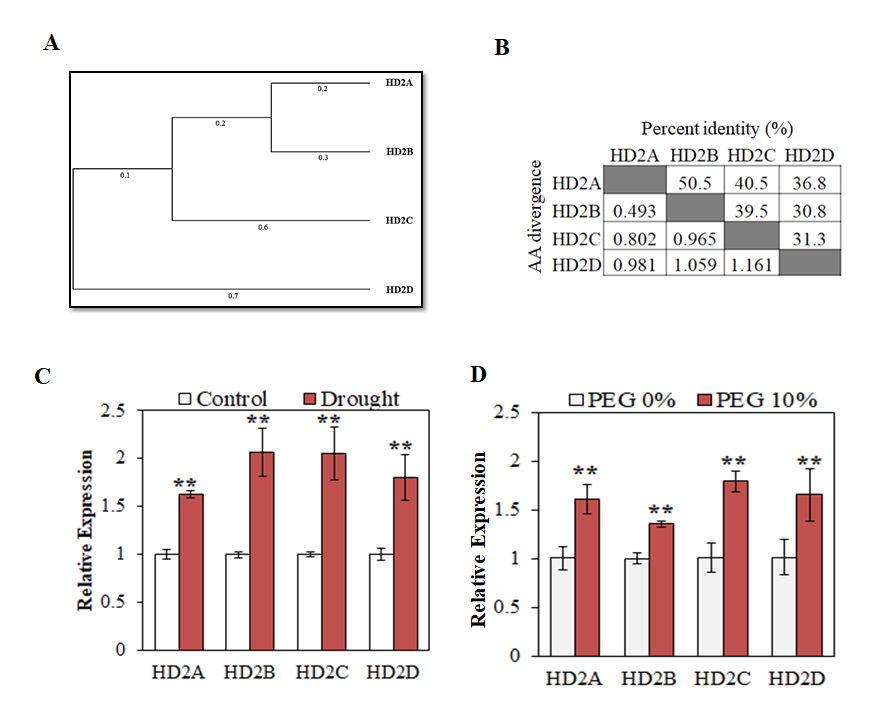


**Figure S1: Phylogenetic analysis of HD2 proteins**

(A) A phylogenetic tree was constructed using protein sequences of Arabidopsis HD2A, HD2B, HD2C and HD2D. (B) Amino acid sequences identity and divergence of the four Arabidopsis HD2 proteins.


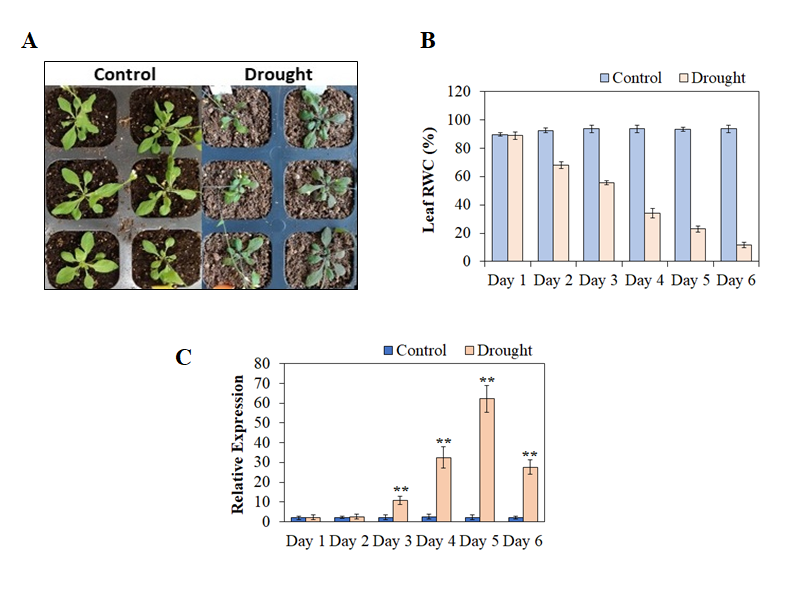


**Figure S2: Drought stress induced changes in Arabidopsis plant**

(A) Images of WT plants under control conditions and soil drought stress at day 6. (B) Leaf relative water content measured under control and stress conditions from day 1 to day 6. Data shown are means ± standard errors (n = 9). Three independent experiments were performed. In each experiment, measurements were taken from three plants (total three leaves per plant weighed). (C) Relative expression of RD29A under control and drought conditions. Data are shown as the expression levels relative to the control condition. Data shown are means ± standard errors (n = 3). The significance of the difference was determined by Student’s t test (**p < 0.01).


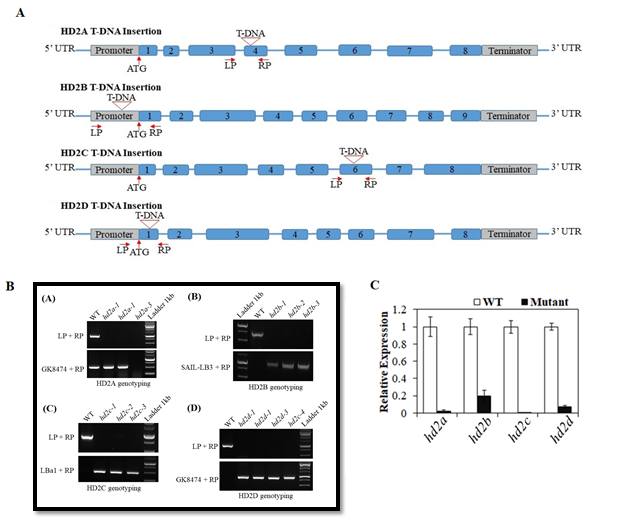


**Figure S3: Confirmation of hd2 TDNA insertion mutants**

(A) T-DNA insertion map of HD2 genes for *hd2* mutant lines. Blue blocks indicate exons. Red triangles indicate the position of T-DNA insertions in the HD2 genomic sequences, whereas horizontal red arrows show primer positions in the sequences. (B) PCR Genotyping of *hd2a*, *hd2b*, *hd2c*, and *hd2d* mutant lines. Gene-specific left (LP) and right (RP) primers along with T-DNA primers (LB) were used in genotyping. LB primers used in the genotyping are GK8474 (for *HD2A* and *HD2D*), LB3 (for *HD2B*), and LBa1 (for *HD2C*). (C) Relative expression of *HD2A*, *HD2B*, *HD2C*, and *HD2D* in *hd2a*, *hd2b*, *hd2c*, and *hd2d* mutants, respectively. Data shown are means ± standard errors (n = 3).


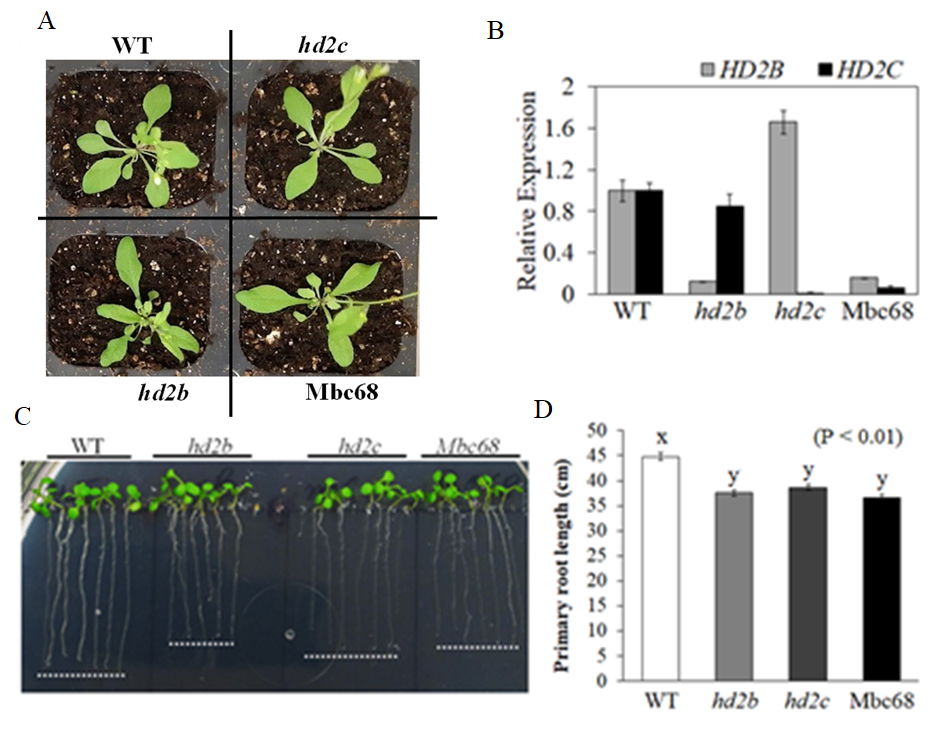


**Figure S4:** **Plant images, gene expression and root growth analysis of *hd2b*, *hd2c*, and Mbc68 mutants.**

(A) Images of 4-week-old WT, *hd2b*, *hd2c*, and Mbc68 plants (B) Relative expression of *HD2B* and *HD2C* in WT, *hd2b*, *hd2c* and Mbc68 plants. Expression of *HD2C* was not affected in *hd2b* mutant whereas the expression of *HD2B* was upregulated in *hd2c* mutant. (C-D) Primary root lengths of 10-day old WT, *hd2b*, *hd2c*, and Mbc68 seedlings under control conditions. Root length in double mutant Mbc68 did not change significantly as compared to the respective single mutants, *hd2b* and *hd2c*. Data shown are means ± standard errors (n = 3). Lowercase letters indicate significant differences (p < 0.01).


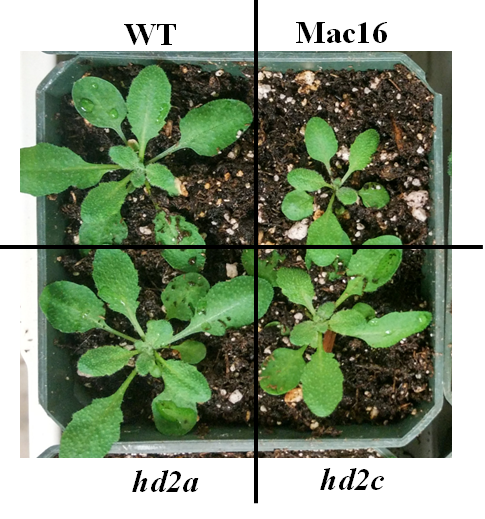


**Figure S5:** Image of 3 week old WT, *hd2a*, *hd2c*, and Mac16 plants.


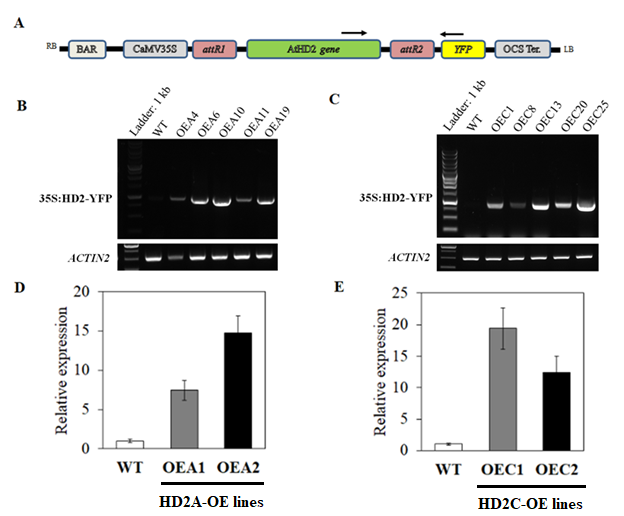


**Figure S6: Generation of HD2 overexpression lines**

(A) Schematic representation of the region of HD2-OE plasmid construct used to generate HD2 overexpression lines. The green box represents the coding region of HD2 genes, under the control of the CaMV35S promoter. The yellow box represents the sequence of the reporter gene, yellow fluorescent protein (*YFP*). RB and LB indicate the right and left border of the T-DNA region, respectively. Black arrows represent the position of primers used for PCR confirmation in transgenic plants. (B-C) Confirmation of the presence of 35S-HD2A-YFP in the HD2A-OE (B) and 35S-HD2C-YFP in the HD2C-OE (C) lines. The genomic DNA of WT Arabidopsis was used as negative control. A fragment of 854 bp of 35S-HD2A-YFP construct in the HD2A-OE lines and 896 bp fragment of 35S-HD2C-YFP in the HD2C-OE lines was amplified using the primer pair indicated by black arrows in (A). *ACTIN2* gene primers were used to amplify the gene to indicate the genomic DNA quality. The homozygous transgenic lines OEA6 and OEA10 for HD2A-OE were selected and renamed as OEA1 and OEA2 for convenience. Similarly, OEC13 and OEC25 were selected for HD2C-OE and renamed as OEC1 and OEC2, respectively. (D-E) Relative expression of *HD2A* (D) and *HD2C* (E) in HD2A-OE and HD2C-OE lines, respectively. Data shown are means ± standard errors (n = 3).

**
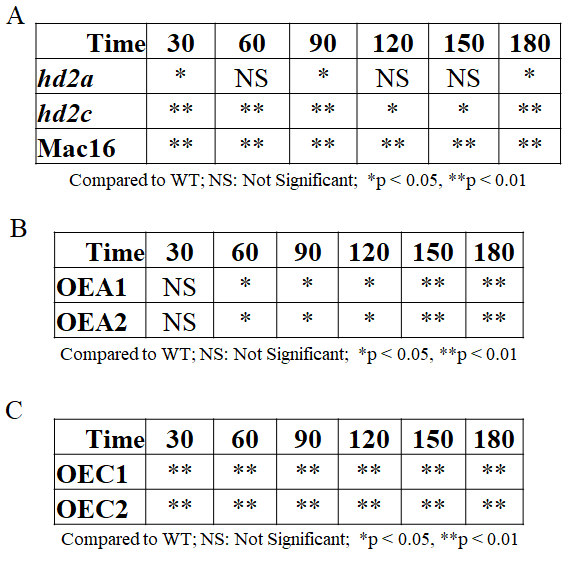
**

**Figure S7: Statistical analysis of fresh leaf water loss experiments**

(A) Statistical significance of fresh leaf water loss measured in WT, *hd2a*, *hd2c*, and Mac16 at every 30-minute interval for the period of 180 minutes. The significance of differences between various genotypes was determined by one-way ANOVA followed by post-hoc Tukey’s HSD tests. (B-C) Statistical significance of fresh leaf water loss measured in HD2A-OE (B) and HD2C-OE (C) at every 30-minute interval for the period of 180 minutes. The significance of the differences between different genotypes was determined by Student’s t test.
