# Supplementary material for: HD2A and HD2C co-regulate drought stress response by modulating stomatal closure and root growth in Arabidopsis
Source: Front Plant Sci. 2022 Nov 23;13:1062722. doi: 10.3389/fpls.2022.1062722 (PMC9727301; doi:10.3389/fpls.2022.1062722)
Supplement: Supplementary file 2 [file DataSheet_2.docx]

**Supplementary File 2: Figure 1-7**

**Original Uncropped Gels and Blots**


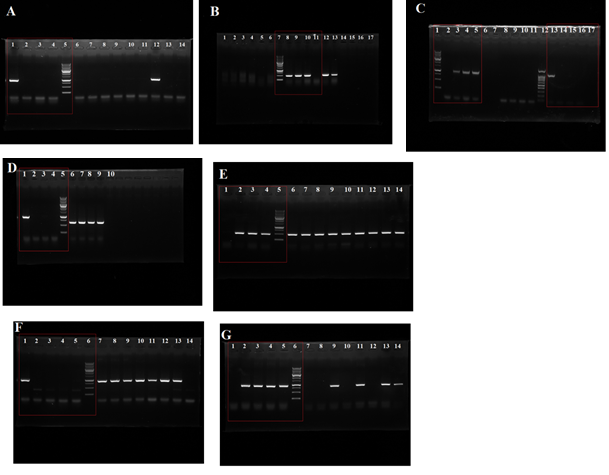


Figure S1: Uncropped gel images of Figure S3B in supplementary data file 1. A-B gel Images correspond to HD2A genotyping, cropped from lanes 1-5 and 7-11 and dragged the ladder to the left*.* C gel Image corresponds to HD2B genotyping, cropped from lanes 1-5 and 13-17*.* D-E gel Images correspond to HD2C genotyping, cropped from lanes 1-5*.*  F-G gel Images corresponds to HD2D genotyping*,* cropped from lanes 1-6.


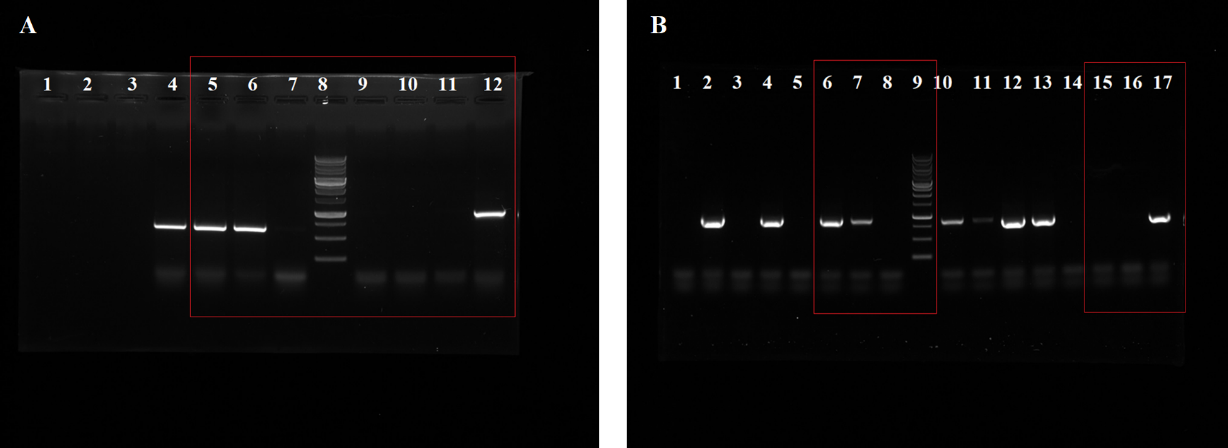


Figure S2: Uncropped gel images of Figure 2A. A gel Image correspond to HD2A genotyping, cropped from lanes 5-11 and dragged the ladder to the left. B gel image correspond to HD2C genotyping, cropped from lanes 6-9 and 15-17 and dragged the ladder to the left*.*


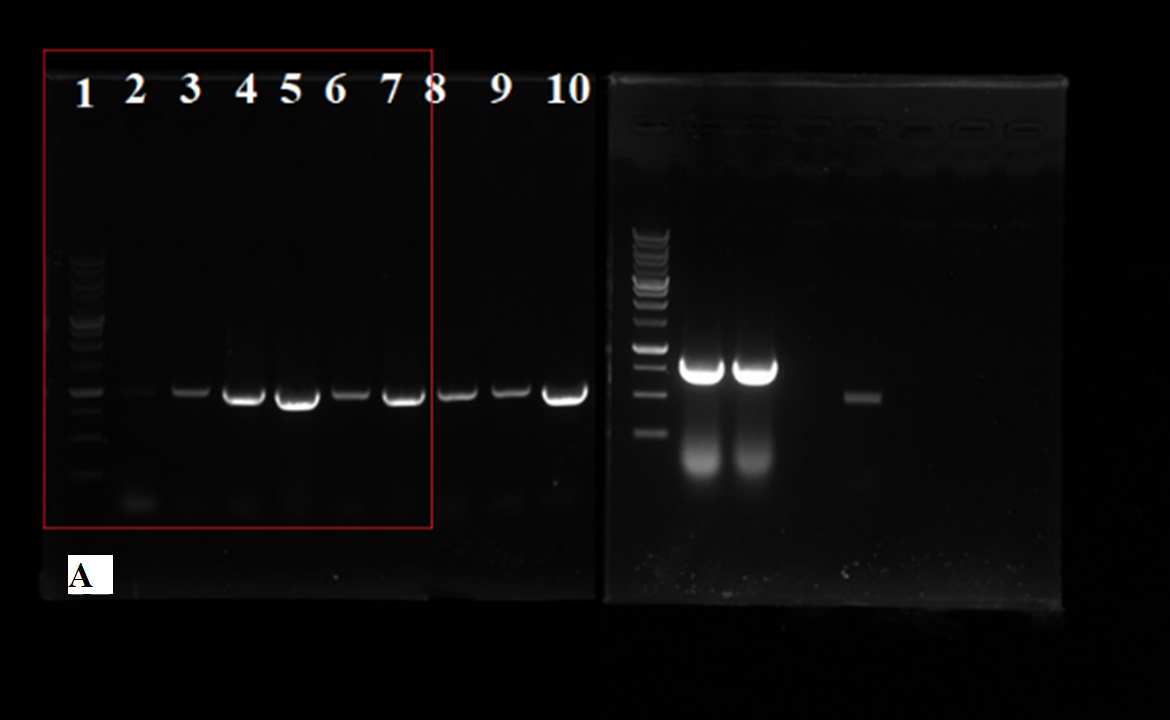

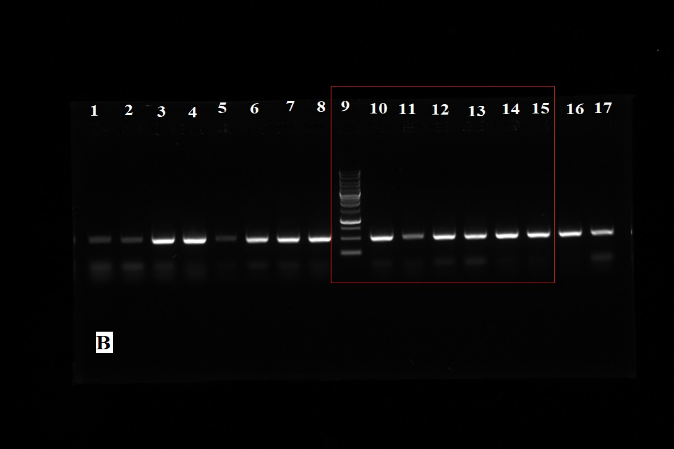


Figure S3: Uncropped gel images of Figure S6B. A gel Image corresponds to amplification of HD2A-YFP by RT-PCR in HD2A-OE lines, cropped from lanes 1-7. B gel image correspond to lower panel of Figure S6B showing ACTIN2 amplification, cropped from lanes 9-15.


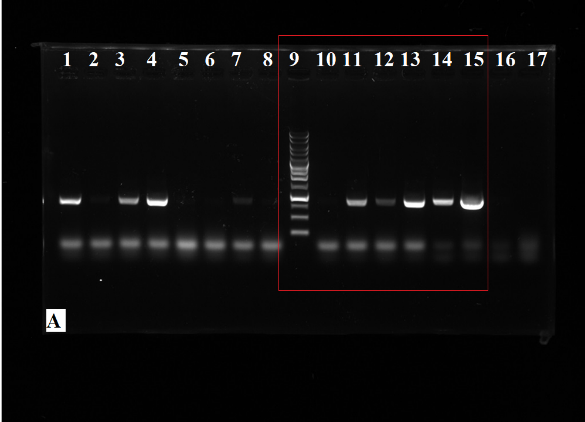

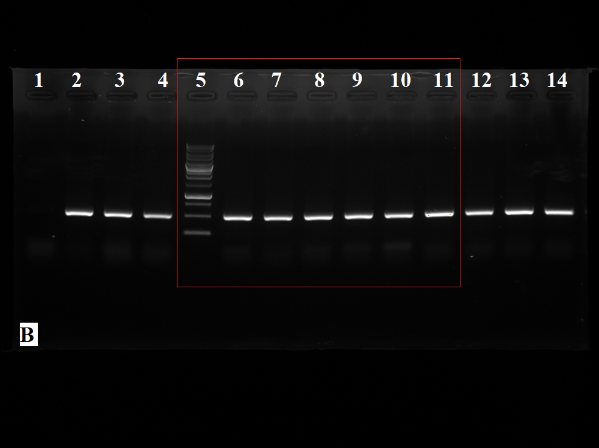


Figure S4: Uncropped gel images of Figure S6C. A gel Image corresponds to amplification of HD2C-YFP by RT-PCR in HD2C-OE lines, cropped from lanes 9-17. B gel image correspond to lower panel of Figure S6C showing ACTIN2 amplification, cropped from lanes 5-11.


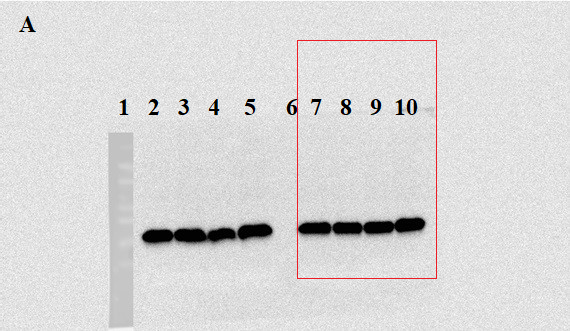

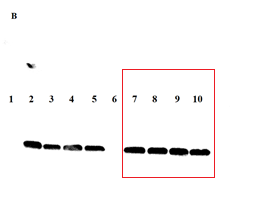


Figure S5: Uncropped western blot images of Figure 8A. A blot Image corresponds to the upper panel of Figure 8A showing H3K9ac levels in *hd2* mutants, cropped from lanes 7-10. B blot image correspond to the lower panel of Figure 8A showing H3 levels, cropped from lanes 7-10.


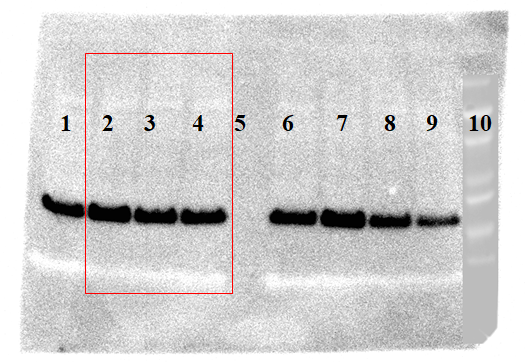

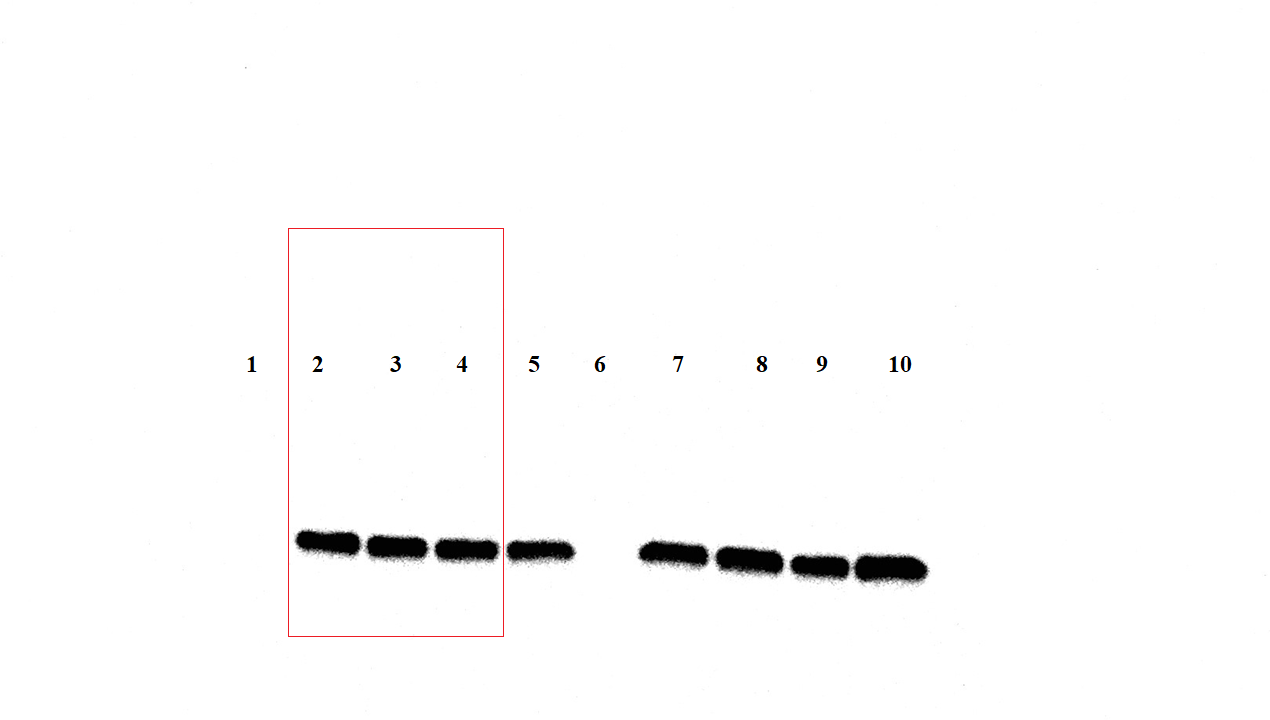


Figure S6: Uncropped western blot images of Figure 8B. A blot Image corresponds to the upper panel of Figure 8B showing H3K9ac levels in HD2A-OE lines, cropped from lanes 2-4. B blot image correspond to the lower panel of Figure 8B showing H3 levels, cropped from lanes 2-4.


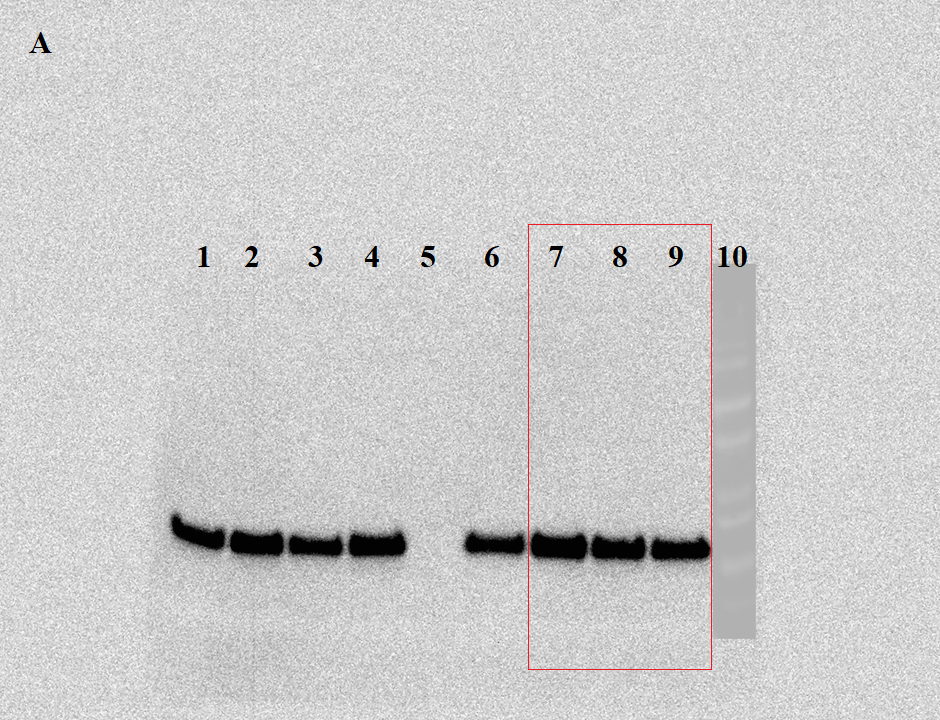

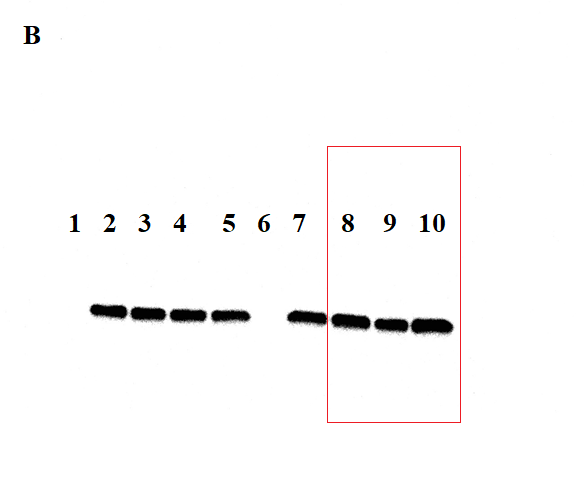


Figure S7: Uncropped western blot images of Figure 8C. A blot Image corresponds to the upper panel of Figure 8C showing H3K9ac levels in HD2C-OE lines, cropped from lanes 7-9. B blot image correspond to the lower panel of Figure 8C showing H3 levels, cropped from lanes 8-10.
