# Supplementary material for: HD2A and HD2C co-regulate drought stress response by modulating stomatal closure and root growth in Arabidopsis
Source: Front Plant Sci. 2022 Nov 23;13:1062722. doi: 10.3389/fpls.2022.1062722 (PMC9727301; doi:10.3389/fpls.2022.1062722)
Supplement: Supplementary file 3 [file DataSheet_3.docx]

**Supplementary File 3 – Tables 1-3**

**Table 1: Primers used for PCR genotyping**

| Genes | Primers | 5` Sequences 3` |
| --- | --- | --- |
| HD2A | HD2A_GK355F | CGGCTTCGTATTAAAACCCTC |
|  | HD2A_GK355R | GCCTTTGGTTTAGCTACAGCC |
| HD2B | HD2B_SL1247F | TCTTCTTCTCCTCCCGATAGC |
|  | HD2B_SL1247R | AACAAGTTTAGCCCCACCAAC |
| HD2C | HD2C_SALK_F | GATGATCAACTTGAGGCTGCT |
|  | HD2C_SALK_R | CAGAATTCTTACCCGCCTGT |
| HD2D | HD2D_GK279F | ACCCTACCTGTGAAAAGGAAG |
|  | 2D_GK279R | CCCATTCTCATCATCACCAATC |
| LB primer | GK8474 | ATAATAACGCTGCGGACATCTACATT |
| LB primer | LB3 | TAGCATCTGAATTTCATAACCAATCTCGATACAC |
| LB primer | LBb1.3 | ATTTTGCCGATTTCGGAAC |
| LB primer | LBa1 | TGGTTCACGTAGTGGGCCATCG |

**Table 2: Primers used for Gateway cloning**

| Genes | Primers | 5` Sequences 3` |
| --- | --- | --- |
| HD2A | AtHD2A_GW_F | GGGGACAAGTTTGTACAAAAAAGCAGGCTACATGGAGTTCTGGGGAATTGAAG |
|  | AtHD2A_GW_R | GGGGACCACTTTGTACAAGAAAGCTGGGTCCTTGGCAGCAGCGTGCTT |
| HD2B | AtHD2B_GW_F | GGGGACAAGTTTGTACAAAAAAGCAGGCTACATGGAGTTCTGGGGAGTTG |
|  | AtHD2B_GW_R | GGGGACCACTTTGTACAAGAAAGCTGGGTCAGCTCTACCCTTTCCCTTG |
| HD2C | AtHD2C_GW_F | GGGGACAAGTTTGTACAAAAAAGCAGGCTACATGGAGTTCTGGGGTGTTG |
|  | AtHD2C_GW_R | GGGGACCACTTTGTACAAGAAAGCTGGGTCAGCAGCTGCACTGTGTTTG |
| HD2D | AtHD2D_GW_F | GGGGACAAGTTTGTACAAAAAAGCAGGCTACATGGAGTTTTGGGGTATCGA |
|  | AtHD2D_GW_R | GGGGACCACTTTGTACAAGAAAGCTGGGTCCTTTTTGCAAGAGGGACCA |
|  |  |  |
|  |  |  |

**Table 3: Primers used for RT-qPCR analysis**

| Genes | Primers | 5` Sequences 3` |
| --- | --- | --- |
| HD2A | HD2A_CDS_F3 | GTGAAGCCAGCTGTTGATGA |
|  | HD2A_CDS_R3 | CTTTCGCCTTCTTTGCTGAC |
| HD2B | HD2B_CDS_F2 | ATCCAAAACACCCGTCTCTG |
|  | HD2B_CDS_R2 | CCGGATGATTGACCTCCAG |
| HD2C | HD2C_CDS_F1 | CAGGTTGCTTTGGGAGAGAG |
|  | HD2C_CDS_R1 | GTATGAGACAGCGCAAAGTTCC |
| HD2D | HD2D_CDS_F1 | AGATGGGTTTGGACGAGGATG |
|  | HD2D_CDS_R1 | GGGCATCTCTTCTTCCCTCC |
| RD29A | RD29A_qF | AACGACGACAAAGGAAGTGG |
|  | RD29A_qR | AACCAGCCAGATGATTTTGG |
| SLAC1 | SLAC1CT_F | TGGAAACAGAGGACCAAACC |
|  | SLAC1CT_F | TCTGTTTTCCGACCATCTCC |
| ACTIN2 | ACT2_RT_FOR | TATCGCTGACCGTATGAGCA |
|  | ACT2_RT_REV | ATCATACTCGGCCTTGGAGA |
| GA2ox1 | Ga2OX1_F | CAAGAGCGTGAGGCATAGGG |
|  | Ga2OX1_R | AGTCAATGAAGGTCCAGCGAAG |
| GA2ox2 | Ga2OX2_F | CATTCTCTGCGGTTTGTTTGG |
|  | Ga2OX2_R | CGTGAGTCTCAGTGTCTACATAG |
| GA2ox3 | Ga2OX3_F | TGCCTGAGAATGAACCATTACCC |
|  | Ga2OX3_R | TGTTCCATCTTTGACACAGATTTGC |
| GA2ox4 | Ga2OX4_F | GCTCGGCAGTGAATTGTTACATAG |
|  | Ga2OX4_R | CACAGATTGGTCAGAAAGATTGGC |
| GA2ox6 | Ga2OX6_F | ACAGAAGTCTAGCGAAGTGAGTG |
|  | Ga2OX6_R | CGGTGCTGGTGGATAGTGATTC |
| ABI1 | ABI1_F | TGAAGAAGCGTGTGAGATGG |
|  | ABI1_R | CTGTATCGCCAGCTTTGACA |
| ABI2 | ABI2_F | GATGGAAGATTCTGTCTCAACGATT |
|  | ABI2_R | GTTTCTCCTTCACTATCTCCTCCG |
